# Onset Potential for Electrolyte Oxidation and Ni-Rich Cathode Degradation in Lithium-Ion Batteries

**DOI:** 10.1021/acsenergylett.2c01722

**Published:** 2022-09-22

**Authors:** Wesley M. Dose, Weiqun Li, Israel Temprano, Christopher A. O’Keefe, B. Layla Mehdi, Michael F. L. De Volder, Clare P. Grey

**Affiliations:** † Department of Chemistry, University of Cambridge, Lensfield Road, CB2 1EW Cambridge, U.K.; ‡ Department of Engineering, University of Cambridge, 17 Charles Babbage Road, CB3 0FS Cambridge, U.K.; § The Faraday Institution, Quad One, Harwell Science and Innovation Campus, Didcot OX11 0RA, U.K.; ∥ Department of Mechanical, Materials and Aerospace Engineering, University of Liverpool, Liverpool L69 3GH, U.K.

## Abstract

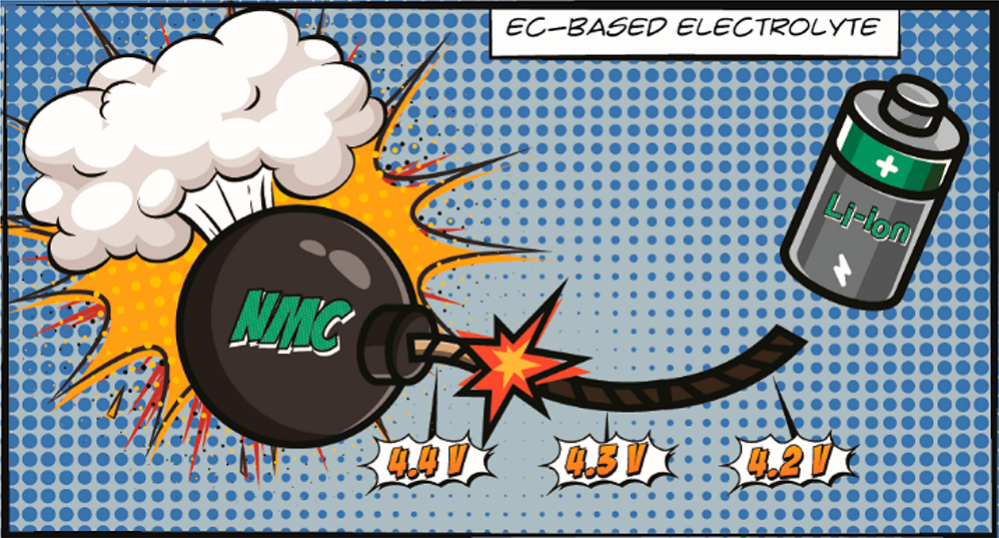

High-capacity Ni-rich layered metal oxide cathodes are highly desirable to increase the energy density of lithium-ion batteries. However, these materials suffer from poor cycling performance, which is exacerbated by increased cell voltage. We demonstrate here the detrimental effect of ethylene carbonate (EC), a core component in conventional electrolytes, when NMC811 (LiNi_0.8_Mn_0.1_Co_0.1_O_2_) is charged above 4.4 V vs Li/Li^+^—the onset potential for lattice oxygen release. Oxygen loss is enhanced by EC-containing electrolytes—compared to EC-free—and correlates with more electrolyte oxidation/breakdown and cathode surface degradation, which increase concurrently above 4.4 V. In contrast, NMC111 (LiNi_0.33_Mn_0.33_Co_0.33_O_2_), which does not release oxygen up to 4.6 V, shows a similar extent of degradation irrespective of the electrolyte. This work highlights the incompatibility between conventional EC-based electrolytes and Ni-rich cathodes (more generally, cathodes that release lattice oxygen such as Li-/Mn-rich and disordered rocksalt cathodes) and motivates further work on wider classes of electrolytes and additives.

Electrochemical energy storage is a key technology in the pursuit of net zero emissions and to mitigate climate change. Lithium-ion batteries (LIBs) are currently the leading storage chemistry in many sectors, including electric vehicles, and are becoming increasingly more important for use in grid-scale storage. Promising next-generation cathode materials with lower cost and higher energy density are being researched intensely with the aim to resolve the rapid performance fading issues that presently limit their lifetime. Increasing the Ni content, as in Ni-rich LiNi_0.8_Mn_0.1_Co_0.1_O_2_ (NMC811), and increasing the upper cutoff voltage (to extract more capacity from the cathode) are two main approaches that are pursued, oftentimes in parallel.^1,2^ Several recent reviews highlight the scientific challenges facing Ni-rich cathodes and the approaches being explored by the research community to mitigate them.^1,2^ The leading cause for capacity fade in LIBs is active (Li^+^) ion loss (AIL) as a result of lithium-immobilizing (electrolyte degradation) reactions that create (in the first few cycles) and thereafter repair the solid electrolyte interphase (SEI) at the graphite anode.^1,2^ Damage caused to the SEI in the course of electrochemical cycling is generally attributed—at least in part—to crossover of species generated at the cathode to the anode. Acidic species, formed by electrolyte oxidation,^3−9^ and dissolved transition metals (TMs)^10−13^ are the two main culprits; both are reported to be more problematic for Ni-rich cathodes.^13,14^

Decomposition of the electrolyte at high potentials can occur by electrochemical oxidation (e.g., >4.95 V vs Li/Li^+^)^6^ or chemical oxidation. (Note that all potentials stated in this work are, or are converted to be, vs Li/Li^+^.) Below the upper cutoff potentials (UCPs) typical for NMC, i.e., <4.6 V, electrolyte breakdown is mostly attributed to chemical oxidation of the electrolyte solvents.^5,14−16^ Release of reactive lattice oxygen from the NMC surface, which coincides with the evolution of CO_2_ and CO, is suggested as the dominant cause for electrolyte oxidation.^5,14−17^ The onset potential for gas release is lower for Ni-rich NMCs (e.g., ∼4.4 V for NMC811 versus ∼4.6 V for LiNi_0.33_Mn_0.33_Co_0.33_O_2_ (NMC111)),^5,15^ which is attributed to the higher degree of lithium extraction achieved at lower potential.^5,15,18^ Oxidative decomposition of the carbonate solvent(s) generates water and other protic species^3−5^ that, in LiPF_6_-based electrolytes, decompose PF_6_^–^ to PF_5_ and HF, with further reactions forming Lewis acidic OPF_3_ and fluorophosphate salts, such as PO_2_F_2_^–^.^6−9^ Such species have been shown to rapidly decompose integral components of the graphite SEI, such as lithium ethylene dicarbonate.^19^ This mechanism has recently been proposed as a leading cause for AIL and capacity loss in LIBs.^9^

TM dissolution from NMC (irrespective of Ni-content) is a long-standing, yet largely unsolved, degradation process in LIBs. Temperature and high UCP are known to amplify dissolution.^10^ It has also been reported to coincide with electrolyte oxidation, rationalized by HF formation (see above) and acid-mediated dissolution.^9,11,13,20−22^ Dissolved TMs migrate through the electrolyte to the graphite anode where they deposit, and have been proposed to cause continuous decomposition of the SEI and electrolyte, associated AIL, and increased anode impedance.^10−12^ Therefore, TM dissolution is directly linked to capacity and power fading.

Recent reports highlight that the electrolyte solvent has a profound impact on Ni-rich NMC (and high-voltage) cell lifetime. Specifically, ethylene carbonate (EC)-free electrolytes (containing a small amount of an SEI former such as vinylene carbonate (VC)) outperform conventional LIB electrolytes, which are a mixture of EC and linear carbonate(s), such as ethyl methyl carbonate (EMC).^23−25^ To understand the fundamental mechanisms behind these observations, we recently showed that while reactive lattice oxygen reacts with both EC and EMC, the lattice oxygen release, electrolyte oxidation, and NMC surface degradation are significantly suppressed with an EC-free electrolyte.^13^ The electrolyte-dependent degradation was analyzed at high NMC potential (i.e., 4.6 V) “stressed” conditions. Importantly, this study identified the critical degradation pathways in EC-containing and EC-free electrolytes; however, the onset potentials for the degradation processes in the two classes of electrolytes are not currently known. This knowledge is an important step toward enabling long cycle life for Ni-rich NMC in LIBs.

In this work, we subject low- and high-Ni NMC to potentiostatic holds at 4.3, 4.4, 4.5, or 4.6 V to determine the onset potential of the important cathode and electrolyte degradation processes with EC-containing and EC-free electrolytes. For Ni-rich NMC, we clearly show that electrolyte oxidation/breakdown and detrimental cathode surface degradation, including surface reconstruction and TM dissolution, are initiated by charging above the ∼4.4 V onset for lattice oxygen release and that the EC component of conventional carbonate-based electrolytes is predominantly responsible. A significantly suppressed degree of degradation is revealed for NMC111 at all potentials tested irrespective of whether the electrolyte has EC, and for NMC811 with EC-free electrolyte, even above 4.4 V. The nature of these effects are uncovered by online electrochemical mass spectrometry (OEMS), electrochemical impedance spectroscopy (EIS), and post-mortem analysis by solution NMR, transmission electron microscopy (TEM), and inductively coupled plasma-optical emission spectroscopy (ICP-OES).

After a slow *C*/20 charge, NMC111 and 811 cathodes are subjected to a 60 h voltage hold (VH) at 4.3, 4.4. 4.5, or 4.6 V in NMC/Li_4_Ti_5_O_12_ (LTO) cells to explore the degradation processes or parasitic reactions that occur in charged electrodes (Figure 1a,b; additional electrochemistry data are shown in Figure S1 and Table S1). No earlier formation cycles were applied. An LTO anode, which has a relatively high and constant lithiation potential of 1.55 V (Figure S2), is used to avoid parasitic currents arising from crossover of electrolyte reduction products formed at the anode to the cathode.^26−28^ The baseline electrolyte used is 1 M LiPF_6_ in a mixture of EC and EMC in a wt. ratio of 3:7, referred to as LP57. This conventional electrolyte is compared against single-solvent electrolytes, 1.5 M LiPF_6_ in EC and 1.5 M LiPF_6_ in EMC (see experimental methods in the Supporting Information), which are used to decouple the effects of cyclic and linear carbonates in conventional electrolytes.

**Figure 1:**
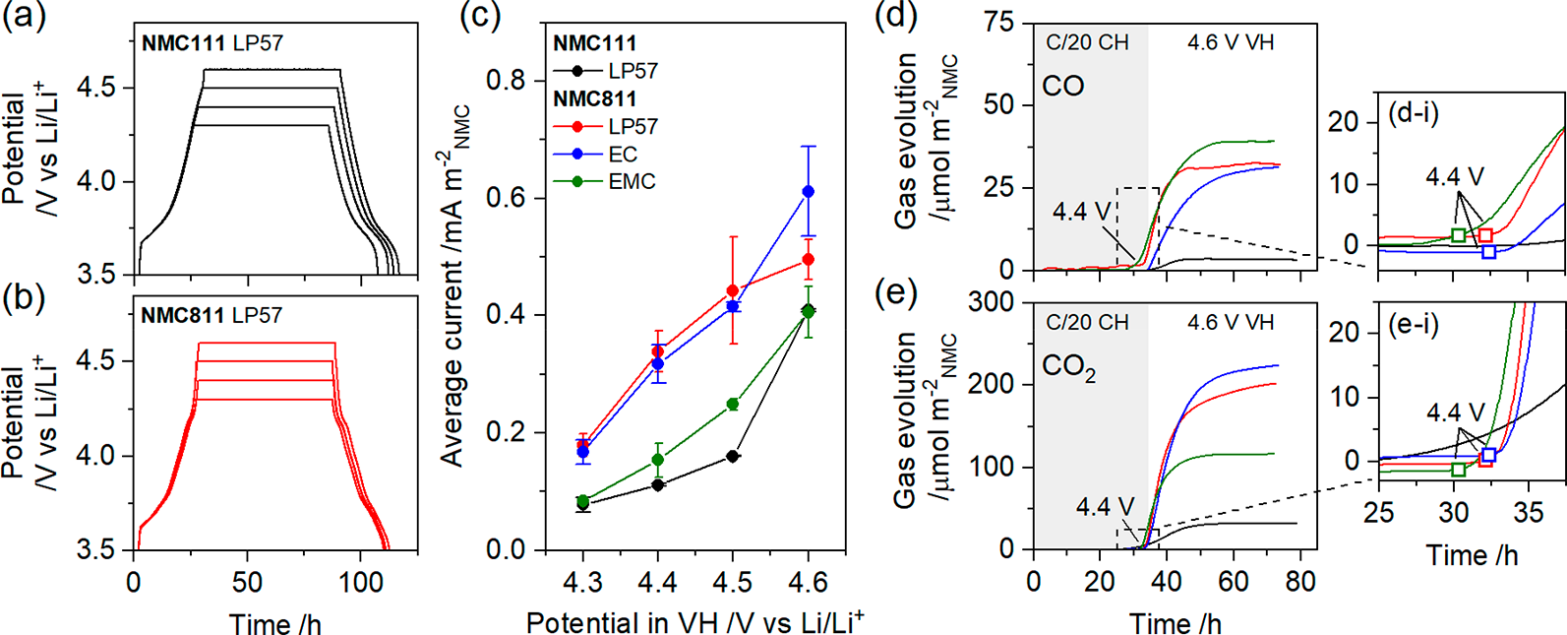
Electrochemistry and gas analysis. (a and b) Representative potential profiles from NMC/LTO cells for (a) NMC111 and (b) NMC811 with LP57 electrolyte during a *C*/20 charge to 4.3, 4.4, 4.5, or 4.6 V, 60 h voltage hold (VH), and *C*/20 discharge. (c) Average current, normalized to the NMC surface area, during the final 20 h of the VH as a function of the NMC potential in the VH with electrolytes LP57, 1.5 M LiPF_6_ in ethylene carbonate (EC), and 1.5 M LiPF_6_ in ethyl methyl carbonate (EMC). Error bars show the spread of at least 2 duplicate cells. (d and e) Evolution of (d) CO and (e) CO_2_ from the OEMS channels *m*/*z* = 28 and 44, respectively, normalized to the NMC surface area, during a *C*/20 charge (CH) and 40 h VH at 4.6 V. The region indicated by the dashed boxes in panels d and e are enlarged in panels d-i and e-i, respectively, which show an onset potential of ∼4.4 V. The colors of the traces in panels d and e are the same as those used in panel c.

In the early stages of the VH, the current decays rapidly (Figure S1) as the electrolyte polarization relaxes and as the NMC particles reach the equilibrium Li^+^ concentration set by the applied potential. The current flowing in the final 20 h of the VH is indicative of the parasitic side reactions taking place at the cathode–electrolyte interface.^13,26,27^Figure 1c shows that the average current, normalized to the NMC surface area (Table S2), increases with higher NMC potential, as expected. However, the current increases more rapidly for NMC811 with EC-containing electrolytes (i.e., LP57 and EC electrolyte) and more gradually for NMC811 with the “EC-free” EMC electrolyte and NMC111 in LP57. The electrolyte-dependent current observed for NMC811 is not evident for NMC111 (Figure S3); that is, the currents measured with EC and EMC electrolyte overlay with that of LP57 and are omitted from Figure 1c for clarity. Clearly the interfacial reactivity is dependent on the applied potential, Ni-content, and, for Ni-rich NMCs, the electrolyte.

The rapid increase in the current >4.4 V corresponds to the onset of lattice oxygen release from NMC811 at ∼4.4 V, inferred from the evolution of CO_2_ and CO in the OEMS experiment (Figure 1d,e and potential profiles for the OEMS experiments in Figure S4) due to chemical oxidation of the electrolyte.^5,14,16,17^ Since oxygen release with EMC electrolyte also starts from ∼4.4 V, we propose that the higher parasitic current at potentials ≥4.4 V with EC-containing electrolytes are caused by enhanced oxygen evolution for these conditions. This hypothesis is supported by the measured current (Figure 1c) since lattice oxygen release contributes to the current, the quantity of CO and CO_2_ evolved (Figure 1d,e and accounting for the reaction stoichiometry; see Supplementary Note S1), and the interfacial structure revealed by HRTEM (see below). The smaller parasitic currents seen at 4.3 V are tentatively ascribed to reactivity (or oxygen loss) at more unstable surfaces, e.g., containing undercoordinated oxygen ions.^29^ While small, the onset of these degradation processes initiates a series of degradation processes, resulting in more rapid capacity fade.^30^

Electrolyte breakdown was further examined by solution NMR of the pristine electrolyte (Figure S5) and electrolyte extracted in the discharged state after the VH. Potential-dependent ^1^H spectra are shown in Figures S6–S8, and the signal assignments are listed in Table S3. The ^1^H spectra reveal an increase in the number of signals from electrolyte solvent breakdown products after VHs > 4.4 V (i.e., aldehyde species from EC, acetal species and methanol from EMC, and poly-ethylene oxide (EO) based oligomers from EC and EMC), consistent with the onset of lattice oxygen release. With EC electrolyte, signals from vinylene carbonate (VC) and fluorophosphate salts are observed at all potentials tested (Figure S7). This observation is consistent with the dehydrogenation of EC to VC on the charged NMC surface, as proposed by Shao-Horn and co-workers,^31^ resulting in protic species on the surface and TM reduction. The protic species can further react with the electrolyte salt (PF_6_^–^), explaining the concomitant observation of fluorophosphate salts.^6−9^ Details of these assignments and the formation mechanisms of the observed species are discussed in detail in our recent work.^13^ Coupled with the oxidative decomposition of the electrolyte is the generation of water and/or acidic species,^3−5^ which are highly detrimental to LIB long-term performance.^9^ This is monitored here by the evolution of H_2_ at the negative (Li) electrode in the OEMS experiment (Figure S9).^3,5,13^ A simultaneous onset potential of H_2_ with CO and CO_2_ (∼4.4 V for NMC811), together with enhanced H_2_ evolution for NMC811 versus 111 with LP57 (1.9 times), provides strong evidence that this is associated with oxygen release from NMC, in accordance with recent findings.^3,5,13^

Oxygen loss from the surface of NMC particles is concomitant with a structural transformation from the initial layered structure to spinel- and/or rock-salt-like structures.^5,32^ The HRTEM micrographs in Figure 2a–d compare the interfacial structure of NMC particles in the discharged (lithiated) state after VHs at 4.3 and 4.6 V. Images of pristine NMC and additional particles sampled at random after VHs are shown in Figures S10–S13. For NMC811 with EC-containing electrolytes (Figure 2b,c), the interfacial structure shows significant differences after VHs at 4.3 versus 4.6 V. Specifically, after a 4.3 V VH, the particle surface structure is mostly layered, with some isolated domains of spinel-like phase (but not on all particles examined, see Figure S13), but a thick (>15 nm) rock-salt-like layer is present after a VH at 4.6 V. In contrast, the changes are less severe for NMC111 (irrespective of electrolyte, Figure S11) and for NMC811 with EMC electrolyte (Figure 2d). For the latter, after a VH at 4.3 V, some particles retain a layered structure while others show a thin (ca. 2–4 nm) surface reconstructed layer with spinel-like and/or rock-salt-like phases, which thickens to ca. 5–8 nm after a 4.6 V VH. Significant conversion from layered (MO_2_) to the oxygen-deficient, rock-salt-like structure (MO) with EC-containing electrolytes at 4.6 V, in contrast with the EC-free electrolyte, provides clear-cut evidence for enhanced oxygen release with EC-containing electrolytes, as argued above.

**Figure 2:**
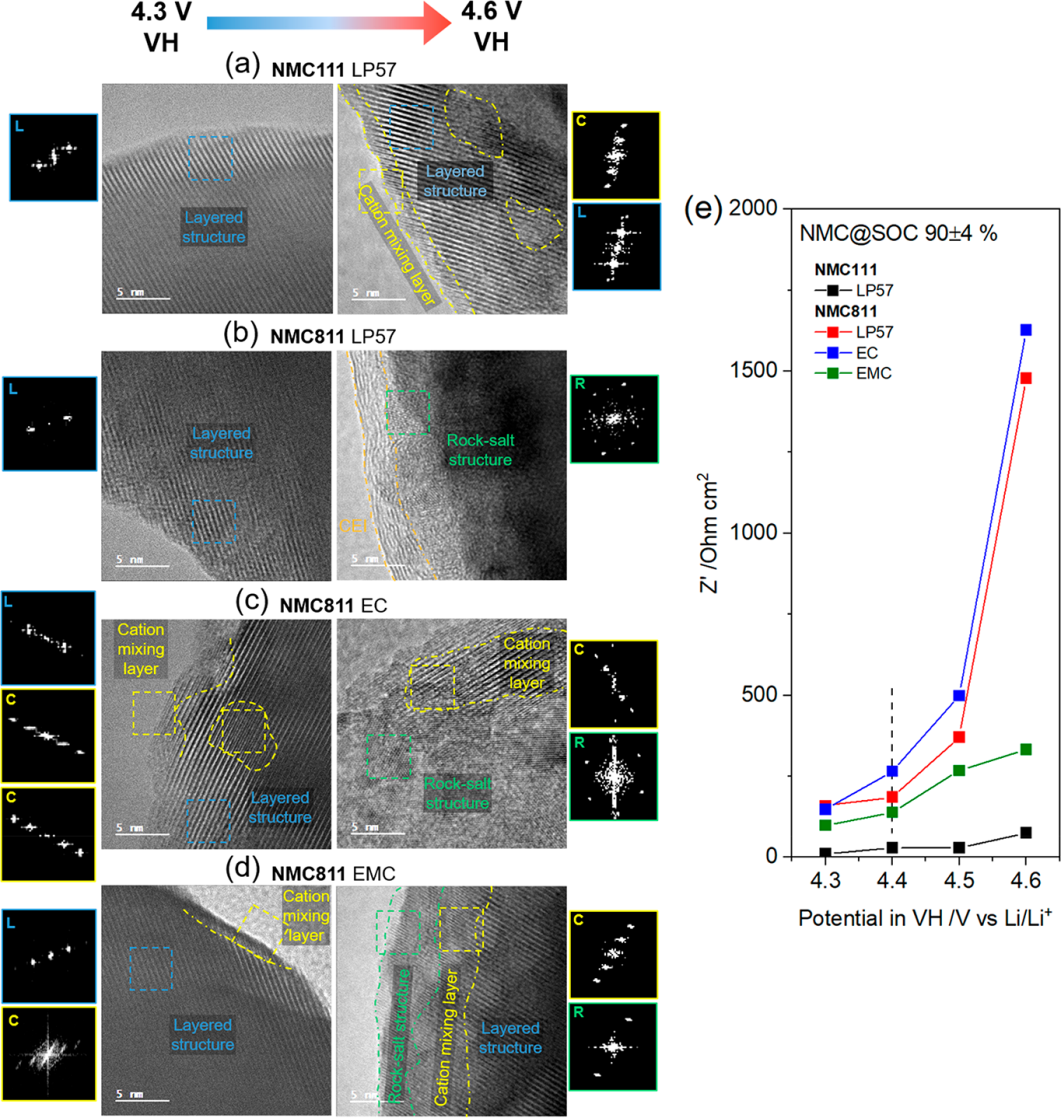
NMC interfacial structure and impedance. (a–d) High-resolution TEM images and corresponding fast Fourier transformation (FFT) images after the voltage holds (VHs) at (left) 4.3 V and (right) 4.6 V for (a) NMC111 with LP57 electrolyte, and NMC811 with electrolytes (b) LP57, (c) 1.5 M LiPF_6_ in EC, and (d) 1.5 M LiPF_6_ in EMC. The dashed squares indicate where the FFTs are analyzed. The letters L, C, and R in the FFTs stand for layered structure, cation mixing layer, and rock-salt structure, respectively. (e) Charge transfer resistance (CTR) from electrochemical impedance spectroscopy (EIS) as a function of NMC potential in the VH, measured at an NMC state of charge (SOC) of 90 ± 4%. The CTR was extracted by fitting a simplified equivalent circuit to the data; see Figure S18 for details. The dashed vertical line indicates the gas evolution onset potential at 4.4 V for NMC811.

The reconstructed layers are poorer Li ion conductors and therefore impede Li transport across this interface,^32,33^ which consequently deteriorates battery performance particularly at high C-rates. Measuring the charge transfer resistance (CTR) using EIS is an easily accessible and rapid approach to quantitatively compare the extent of degradation related to surface reconstruction. After the VH, the CTR of NMC is measured at several potentials/SOCs (using a three-electrode cell), but for clarity only the CTR at 90% SOC is plotted in Figure 2e; associated electrochemical data, details on the data fitting, and the CTR at additional potentials/SOCs are provided in Figures S14–S19. The use of a fixed SOC, rather than a potential, is important when comparing NMCs with different compositions, to ensure they are at the same degree of delithiation. For each condition, the CTR at 90% SOC increases with the VH potential. However, above 4.4 V the CTR of NMC811 increases rapidly with EC-containing electrolytes (reaching ∼1500 Ω cm^2^ after a 4.6 V VH), which is clearly linked to the ∼4.4 V onset potential for gas release from NMC811, discussed above. In contrast, the increase in CTR is more gradual for NMC811 with EMC electrolyte (reaching 333 Ω cm^2^), while much less change is seen for NMC111 (<80 Ω cm^2^) regardless of electrolyte (Figure S19). While the onset potential for gassing is clearly important, the noticeable difference in impedance for NMC811 with EC-containing versus EC-free electrolytes demonstrates that the extent of gassing (i.e., oxygen loss) is a determining factor for impedance-related surface degradation.

Reduced TMs in the surface reconstructed layer (largely TM^2+^) and under coordinated TMs from oxygen loss are probable drivers for dissolution of TMs from the cathode. As such, ICP-OES was conducted to quantify the amount of TM dissolution/deposition as a function of VH potential. As seen in Figure 3, after a VH at 4.3 V the dissolution of Ni, Mn, and Co from NMC811 with all tested electrolytes is low (<5.3, <2.2, and <0.6 ppm, respectively) and on par with NMC111. However, above 4.4 V TM dissolution from NMC811 increases, coinciding with the onset of oxygen release. Dissolution at 4.5 and 4.6 V is most severe with EC electrolyte, followed by LP57. Eliminating EC from the electrolyte, i.e., with EMC electrolyte, leads to significantly suppressed dissolution, even above 4.4 V. The suppressed oxygen release with EMC versus EC electrolyte, and the associated lower generation of water and/or acidic species from electrolyte oxidation, is likely a key contributor to this effect.

**Figure 3:**
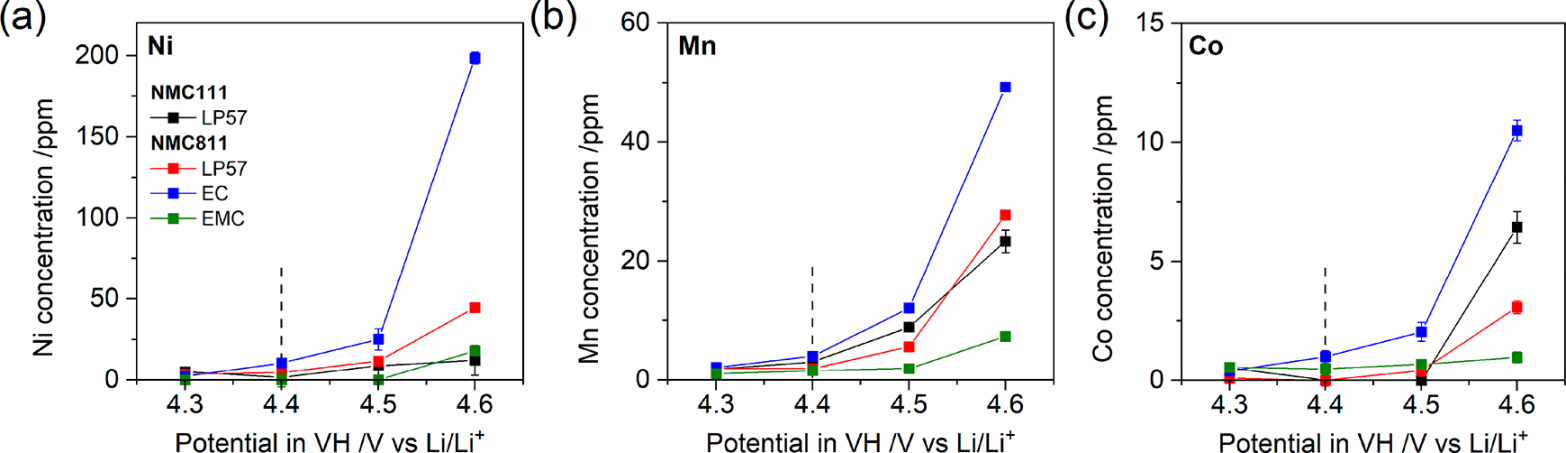
Transition metal dissolution/deposition. Concentration of (a) Ni, (b) Mn, and (c) Co dissolved in the electrolyte and deposited on LTO electrodes, extracted after the VHs in the discharged state. Dashed vertical lines indicate the gas evolution onset potential at 4.4 V for NMC811.

The disparity in TM dissolution from NMC811 with EC electrolyte versus LP57, despite the similar gas evolution (Figure 1d,e), interfacial structure (Figure 2b,c), and impedance (Figure 2e), is proposed to be due to the higher relative stabilizing influence of EC and/or LiPF_6_/EC-based electrolyte degradation products in solvating TMs in solution.^34,35^ Differences in TM dissolution from NMC111 at high potential with EC-containing and EC-free electrolyte (Figure S20), despite much lower gas evolution from NMC111 (Figure 1d,e) and equivalent parasitic currents (Figure S3), further supports a contribution from solvent effects, which is independent of oxygen release.

A schematic illustrating the key degradation pathways proposed in this work is shown in Figure 4. Conventional EC-containing and model EC-free electrolytes display similar behavior below the onset potential of lattice oxygen release from NMC cathodes (left panel), i.e., 4.4 V vs Li/Li^+^ for NMC811. However, above the onset of oxygen release, Ni-rich NMC cathodes with EC-containing electrolytes exhibit more oxygen loss (than with EC-free electrolyte), which in turn leads to more electrolyte breakdown, NMC surface reconstruction, impedance rise, and TM dissolution (middle panel). These degradation processes are shown to be significantly suppressed with EC-free electrolytes (right panel), although eliminating EC does not appear to alter the onset potential of oxygen release. This suggests that oxygen loss is a chemical process that follows the electrochemical Li^+^/e^–^ abstraction, with the coordination strength between the solvent and the cathode surface playing a defining role in the extent of oxygen release.

**Figure 4:**
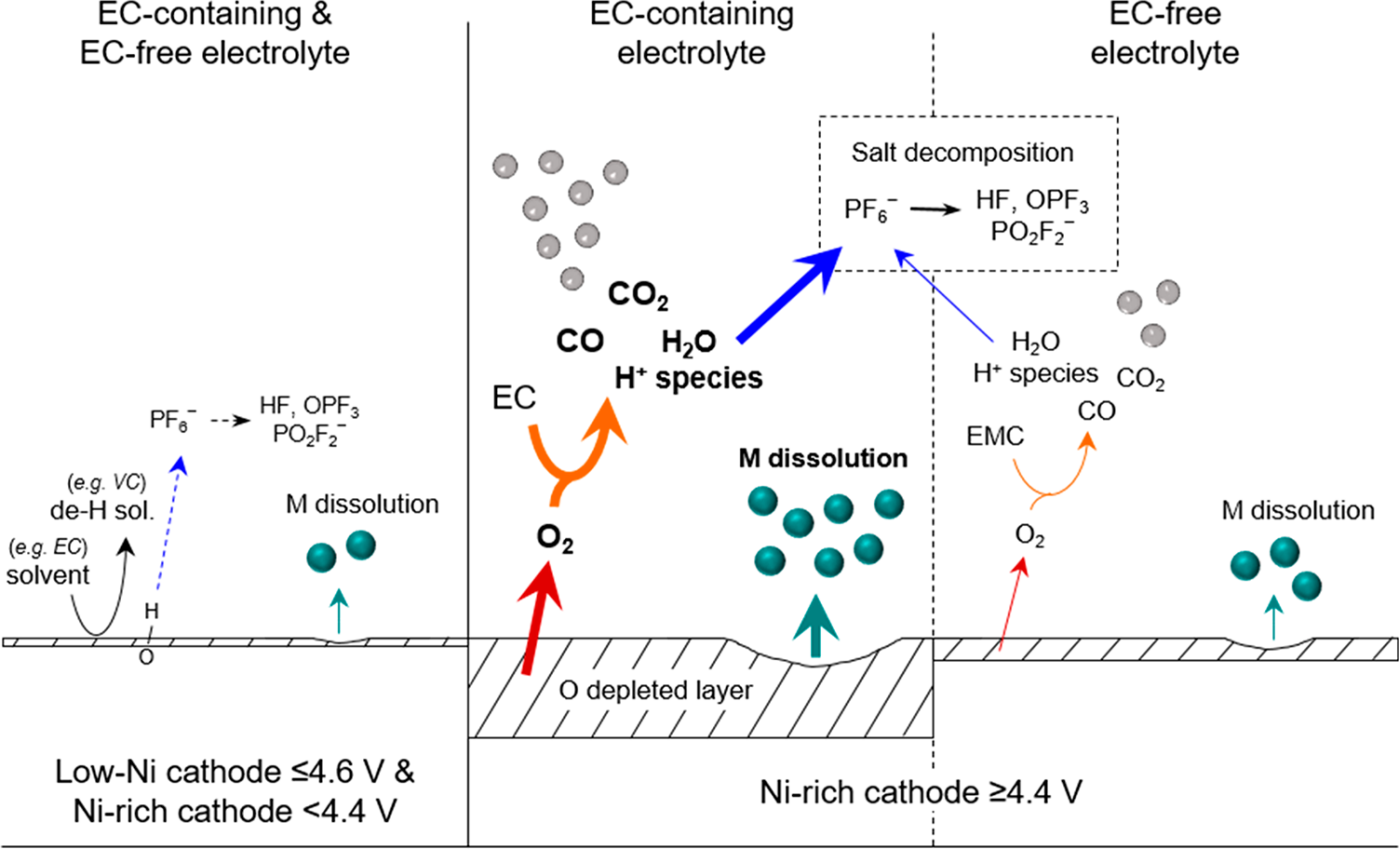
Schematic illustration of the proposed degradation pathways for low-Ni and Ni-rich cathodes with EC-containing and EC-free electrolytes. The image highlights the strong dependence of the electrolyte on the extent of degradation with Ni-rich cathodes charged above the onset potential of lattice oxygen release (4.4 V).

Therefore, while conventional electrolytes are compatible with low-Ni NMC, the EC component poses significant challenges to the cycling stability of high-Ni cathodes. This highlights the conflicting electrolyte needs of Ni-rich cathodes and LIB anodes, whether graphite or next-generation alternatives like silicon and lithium metal/“anode-free”. In conventional LIB electrolytes, EC plays a vital role in the formation and repair of the anode SEI and in inhibiting severe gassing in the case of Li plating on the anode. However, EC is a “bad actor” in the presence of an oxygen-releasing cathode. While solutions to this apparent paradox are yet to be rigorously explored, it is clear from this work that electrolyte development has the potential to enable a step change in the battery performance. The need to suppress oxygen release from the cathode, as highlighted herein, also suggests that materials-based solutions may be an important piece of the puzzle. It is hoped that this work encourages researchers in the field to explore novel electrolyte solutions, material coatings/dopants, and particle morphologies that suppress, or even inhibit, oxygen release—functionality that will surely lead to long cell lifetimes for high-capacity Ni-rich LIBs, as well as other advanced battery chemistries.

## Supporting Information Available

Experimental methods, additional electrochemical data, BET surface area data, additional OEMS data, a note regarding the proposed reactions for the chemical oxidation of EC and EMC, ^1^H NMR spectra and assignments, additional TEM images, and additional ICP data (PDF)
