# Supplementary material for: Onset Potential for Electrolyte Oxidation and Ni-Rich Cathode Degradation in Lithium-Ion Batteries
Source: ACS Energy Lett. 2022 Sep 22;7(10):3524–30. doi: 10.1021/acsenergylett.2c01722 (PMC9578037; doi:10.1021/acsenergylett.2c01722)
Supplement: Supplementary file 1 — nz2c01722_si_001.pdf [file nz2c01722_si_001.pdf]

Supplementary information

Onset potential for electrolyte oxidation and Ni-rich cathode degradation in lithium-ion batteries

Wesley M. Dose,[1,2,3] Weiqun Li,[3,4] Israel Temprano,[1] Christopher A. O'Keefe,[1,3] B. Layla Mehdi,[3,4] Michael F. L. De Volder,[2,3,*] and Clare P. Grey[1,3*]

[1]Department of Chemistry, University of Cambridge, Lensfield Road, Cambridge, CB2 1EW, Cambridge, UK.

[2]Department of Engineering, University of Cambridge, 17 Charles Babbage Road, CB3 0FS, Cambridge, UK.

[3]The Faraday Institution, Quad One, Harwell Science and Innovation Campus, Didcot OX11 0RA, UK.

[4]Department of Mechanical, Materials and Aerospace Engineering, University of Liverpool, Liverpool L69 3GH, UK.

*Correspondence: Clare P. Grey (cpg27@cam.ac.uk), Michael F. L. De Volder (mfld2@cam.ac.uk).

Experimental methods

Electrode fabrication

The NMC111 and NMC811 electrodes contained 90 wt. % NMC (NMC111, Toda; NMC811, Targray), 5 wt. % polyvinylidene difluoride binder (PVDF; Solvay 5130), and 5 wt. % conductive carbon (Timcal C45), and were coated on 20 μm thick aluminum foil using N-methyl-2-pyrrolidone (NMP) as the solvent. The LTO electrodes contained 87 wt. % $Li_4Ti_5O_{12}$ (LTO; Samsung Fine Chemicals), 5 wt. % conductive carbon (Timcal C45), and 8 wt. % PVDF binder (Kureha 9300), and were coated on 20 μm thick aluminum foil using NMP as the solvent. These electrodes were provided by the Cell Analysis, Modeling, and Prototyping (CAMP) Facility at Argonne National Laboratory. NMC111 and NMC811 cathodes were punched to 14 mm diameter and had loadings of 10.10 mg_{NMC111} cm^{-2} (~1.48 mAh cm^{-2} based on 147 mAh g^{-1}_{NMC111}) and 8.21 mg_{NMC811} cm^{-2} (~1.52 mAh cm^{-2} based on 185 mAh g^{-1}_{NMC811}), respectively. LTO anodes were punched to 15 mm diameter and had a loading of 12.27 mg_{LTO} cm^{-2} (~1.96 mAh cm^{-2} based on 160 mAh g^{-1}_{LTO}). The electrode capacity balancing of anode and cathode (N:P ratio) was set to ≈1.3:1.0. Electrodes were calendered to 30 % porosity and

dried at 120 °C for at least 12 h under dynamic vacuum before being transferred to an Ar filled glove box (<0.5 ppm O_2 and H_2O, MBraun).

Full cell experiments

Coin cells (2032-type; Hohsen) were assembled with a NMC cathode versus a LTO anode with a pre-dried 16 mm diameter Celgard 2325 separator (PI-KEM) or glass fiber separator (grade GF/A, Whatman) soaked in 40 µL or 80 µL, respectively, of various electrolytes: 1 M $LiPF_6$ in ethylene carbonate (EC):ethyl methyl carbonate (EMC) 3:7 (by weight, LP57; SoulBrain); 1.5 M $LiPF_6$ (99.9 %; Solvionic) in EC (anhydrous 99 %; Sigma-Aldrich); or 1.5 M $LiPF_6$ in EMC (99.9 %; Solvionic). A 1.5 M salt concentration was used for the EMC and EC electrolytes to improve the ionic conductivity[1] and to lower the viscosity, respectively.[2]

For ICP-OES analysis of the cycled electrolyte, NMC/LTO coin cells were constructed with either three separators (Celgard, glass fiber, Celgard; for LP57 and EMC electrolyte) or one separator (glass fiber; for EC electrolyte) soaked in 120 µL of various electrolytes.

Three-electrode PAT cells (EL-Cell) were assembled with 18 mm diameter cathode and anode, a glass fiber separator (260 µm thickness, grade GF/A) soaked in 100 µL of various electrolytes, and a lithium metal ring electrode set in an insulation sleeve (EL-Cell).

After assembly the cells were cycled in a 25 °C environmental chamber with a Biologic VMP3 potentiostat or BCS 805 series battery cycler. The cycling protocol involved a C/20 (assuming a practical capacity of 147 mAh g^{-1}_{NMC} for NMC111 and 185 mAh g^{-1}_{NMC} for NMC811) charge to various upper cutoff voltages (UCVs) – 2.75, 2.85, 2.95, or 3.05 V (vs. LTO) – a 60 h voltage hold (VH) at the UCV, and a C/20 discharge to 1.45 V. The UCVs correspond to NMC potentials of 4.3, 4.4, 4.5, and 4.6 V vs Li/Li^+, respectively, since the potential of the LTO intercalation plateau is at 1.55 V vs Li/Li^+ (refer to Figure S2). Two or more cells were evaluated for each condition to ensure reproducibility.

Impedance measurements

After the VH protocol the potential-dependent impedance of the NMC cathode was measured in three-electrode PAT cells. The NMC cathode was charged at C/20 to various potentials vs. the Li/Li^+ reference electrode – 3.8, 4.1, 4.3, 4.5, 4.6 V for NMC111 and NMC811, and also 4.7, 4.8, and 4.9 V for NMC111 – held at each potential for 1 h to reach a steady-state, and left to rest at OCP for 1 h before the electrochemical impedance spectroscopy (EIS) measurement. EIS was conducted with a Biologic VMP3 potentiostat in a frequency range of 500 kHz to 10

mHz with an AC voltage perturbation of 5 mV. The SOC of NMC at each potential was calculated based on the charge passed and a theoretical capacity of 277.9 mAh g^{-1}_{NMC} for NMC111 and 275.5 mAh g^{-1}_{NMC} for NMC811.

Online electrochemical mass spectrometry (OEMS)

The OEMS system and the process used to calibrate the MS has been described in detail in ref.[3]. Swagelok-type cells for OEMS were assembled with a NMC cathode (18 mm diameter) versus a lithium metal counter/reference electrode (15.6 mm diameter, 0.25 mm thickness; LTS Research Laboratories) with a glass fiber separator (25 mm diameter, grade GF/B; Whatman) soaked in 300 μL of various electrolytes. After assembly the cells were connected to the OEMS system and allowed to rest at OCP for 6 h before undergoing a cycling protocol involving a C/20 charge to 4.6 V vs. Li/Li^+, a 60 h VH at 4.6 V, a C/20 discharge to 2.5 V vs. Li/Li^+, and an OCP rest period of at least 12 h.

Surface area analysis

The surface area of the NMC powders was determined by nitrogen gas physisorption at 77 K, measuring isothermally at 10 points between $0.07 \leq p/p_0 \leq 0.30$ (3Flex, Micromeritics).

Pristine electrolyte characterization

The water content of the pristine electrolytes was measured by Karl Fischer titration (899 Coulometer, Metrohm). Pristine electrolyte was prepared for NMR analysis by pipetting 40 μL of the electrolyte solution into 0.7 mL of DMSO-d_6 (99.9 atom % D, 99 % CP; Sigma-Aldrich), which was transferred to an airtight NMR tube fitted with a Young's tap.

Sample preparation for post-mortem characterization

After the cycling protocol the NMC/LTO coin cells were disassembled in an Ar filled glove box. The separator was extracted and soaked in 0.7 mL of DMSO-d_6 for 5 min, after which the solution was transferred to an airtight NMR tube fitted with a Young's tap for NMR measurement. The NMC cathodes were extracted, rinsed with 1 mL of dimethyl carbonate (DMC, anhydrous; Sigma-Aldrich), and vacuum dried at ambient temperature for 1 h. After drying, the NMC electrode materials (including NMC111 or NMC811 particles, conductive carbon, and PVDF) were scratched from the aluminum current collector and ground with an agate pestle and mortar. The resulting powder was transferred onto the holey carbon copper grid for TEM.

The NMC/LTO coin cells built for ICP-OES analysis of the cycled electrolyte were dissembled in an Ar filled glove box. The LTO anode and separators were extracted, placed in a 15 mL polypropylene tube, and centrifuged at 5000 rpm for 10 min. The LTO anode and the separators were removed and 4.0 mL of ~2 % nitric acid (diluted from concentrated nitric acid; 67-69 %, trace metal grade; Fischer Chemical) was added to the extracted electrolyte before analysis. The LTO coating was scraped from the current collector and soaked in 248 μL of 5.1 M nitric acid for 5 days before being diluted to 4.0 mL with 3.75 mL of water for analysis. The LTO coating scraped from uncycled electrodes was measured as a baseline.

Solution NMR

NMR experiments were conducted on a Bruker Avance III HD spectrometer equipped with a 11.7 T magnet (ν_{1H} = 500 MHz) using a BBO probe. ^{1}H NMR spectra were acquired using Bloch decay pulse sequence. ^{1}H chemical shifts were referenced to the DMSO-d_6 solvent peak at 2.50 ppm.

ICP-OES

Elemental analysis was performed using inductively coupled optical emission plasma spectroscopy (ICP-OES; Thermoscientific) calibrated with standards prepared from an ICP multi-element solution (VWR, Aristar).

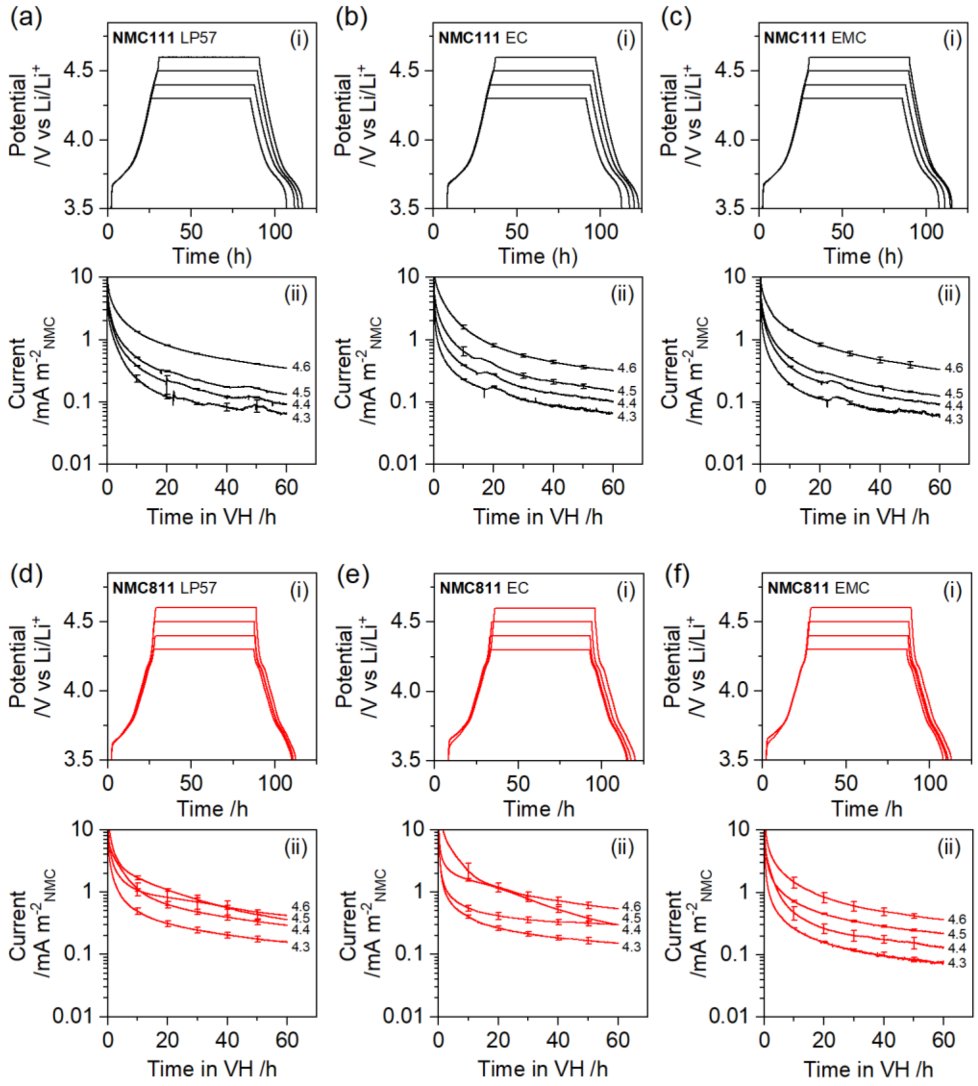

Figure S1. (i) Representative potential profiles during a C/20 charge to 4.3, 4.4, 4.5, or 4.6 V, 60 h voltage hold (VH), and C/20 discharge, and (ii) current during the VH, normalized to the NMC surface area, from NMC/LTO cells for (a-c) NMC111 and (d-f) NMC811 with electrolytes (a, d) LP57, (b, e) 1.5 M $LiPF_6$ in ethylene carbonate (EC), and (c, f) 1.5 M $LiPF_6$ in ethyl methyl carbonate (EMC). Error bars show the spread of at least 2 duplicate cells.

Table S1. Specific capacity values for the charge, voltage hold (VH), and discharge of NMC111 and NMC811 with upper cutoff potentials (UCPs) 4.3, 4.4, 4.5, and 4.6 V vs. Li/Li^+ and with electrolytes LP57, 1.5 M $LiPF_6$ in ethylene carbonate (EC), and 1.5 M $LiPF_6$ in ethyl methyl carbonate (EMC). The numbers in the parenthesis represent the spread of at least 2 duplicate cells.

NMC	UCP /V	Electrolyte	Charge specific capacity /mAh g^{-1}_{NMC}	VH specific capacity /mAh g^{-1}_{NMC}	Discharge specific capacity /mAh g^{-1}_{NMC}
NMC111	4.3	LP57	173.7(4)	5(1)	161.4(5)
		EC	173.2(1)	4.7(1)	158.8(1)
		EMC	176.3(8)	4.4(1)	163.8(5)
	4.4	LP57	191.0(2)	7.6(1)	178.0(2)
		EC	190.5(5)	7.6(1)	175.2(4)
		EMC	190(1)	7.5(1)	177.5(9)
	4.5	LP57	205(1)	9.6(1)	186(1)
		EC	204.2(3)	12(2)	186(2)
		EMC	204.2(4)	9.5(1)	186.3(2)
	4.6	LP57	210(2)	21.9(3)	194(2)
		EC	214.9(2)	25(2)	193.5(1)
		EMC	208(2)	22.5(6)	193.5(9)
NMC811	4.3	LP57	233(3)	7(1)	213(4)
		EC	226(5)	5.7(6)	210(4)
		EMC	224.2(2)	3.9(3)	209(1)
	4.4	LP57	237(2)	13(3)	217(3)
		EC	233(1)	7.9(6)	217(1)
		EMC	228(3)	7(4)	218(3)
	4.5	LP57	242(2)	14(1)	222(3)
		EC	241(3)	16.1(1)	224(3)
		EMC	236.2(6)	9.2(8)	224(1)
	4.6	LP57	250(4)	19.8(8)	230(3)
		EC	254(10)	24(4)	229(6)
		EMC	247(5)	16(2)	228(5)

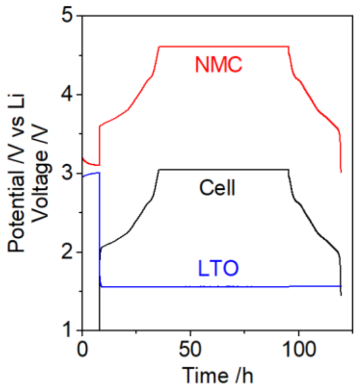

Figure S2. Representative potential profiles of the NMC811 cathode and LTO anode in a three-electrode cell with a Li metal reference electrode and LP57 electrolyte during a C/20 charge to 3.05 V (cell voltage), 60 h voltage hold (VH), and C/20 discharge.

Table S2. Surface area of NMC powders.

NMC	BET surface area /m^2 g^{-1}
NMC111	0.435
NMC811	0.275

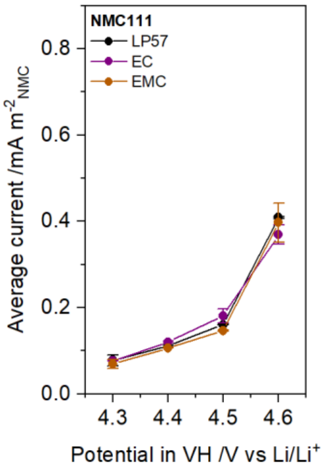

Figure S3. Average current, normalized to the NMC surface area, during the final 20 h of the voltage hold (VH) as a function of the NMC potential in the VH for NMC111 with electrolytes LP57, 1.5 M $LiPF_6$ in ethylene carbonate (EC), and 1.5 M $LiPF_6$ in ethyl methyl carbonate (EMC).

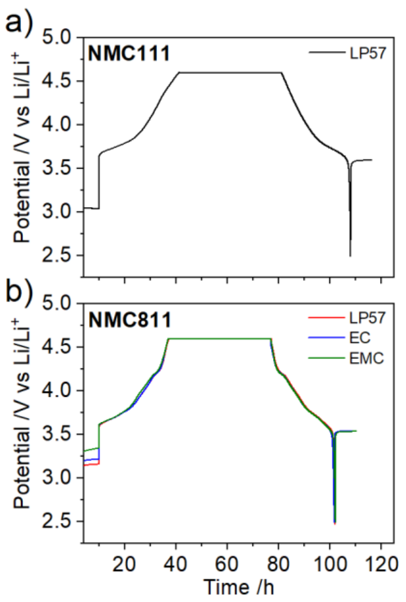

Figure S4. Potential profiles for the NMC/Li OEMS cells during a C/20 charge to 4.6 V, 40 h voltage hold (VH), and C/20 discharge with (a) NMC111 and LP57, and (b) NMC811 and electrolytes LP57, 1.5 M $LiPF_6$ in ethylene carbonate (EC), 1.5 M $LiPF_6$ in ethyl methyl carbonate (EMC).

Supplementary note S1. Proposed reactions for the chemical oxidation of ethylene carbonate (EC) and ethyl methyl carbonate (EMC) and the implications for the interpretation of the quantity of CO and CO_2 evolved.

The proposed reactions for the chemical oxidation of EC and EMC are shown in Scheme S1 and S2, respectively, below.

Scheme S1. Proposed reaction for the chemical oxidation of EC.[4]

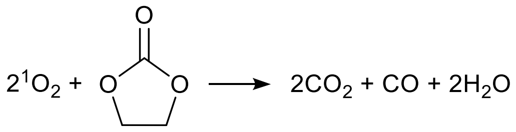

Scheme S2. Proposed reaction for the chemical oxidation of EMC.[3]

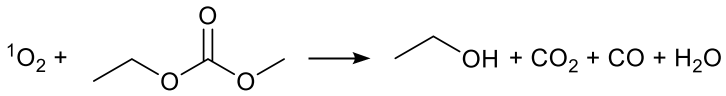

These reactions reveal that the expected O_2:CO_2 mole ratio is 1:1 for both EC and EMC. Therefore, the quantity of CO_2 evolved with EC electrolyte and EMC electrolyte can be directly compared as an indicator for the quantity of (reactive) lattice oxygen release.[3] For CO_2 we measure an EC/EMC fraction of 1.9 (calculated from the data in Figure 1e), suggesting that chemical oxidation of the electrolyte solvent is approx. 1.9 times higher with EC compared to EMC.

For CO, these reactions reveal that the O_2:CO ratio is 2:1 for EC and 1:1 for EMC. In this case, the reaction stoichiometry needs to be taken into account before drawing conclusions about the relative amount of (reactive) oxygen release. Specifically, for the same amount of O_2 release, we would expect the ratio of CO evolved for EC relative to EMC to be 0.5 – i.e. half as much CO would be evolved with EC electrolyte compared to EMC electrolyte. Instead, we measure an EC/EMC fraction of 0.8 (calculated from the data in Figure 1d). This indicates that the chemical oxidation of the electrolyte solvent is approx. 1.6 times higher (0.8/0.5 = 1.6) with EC compared to EMC.

Therefore, the quantity of CO and CO_2 evolved (Figure 1d-e) both indicate that the chemical oxidation of the electrolyte solvent is higher with EC compared to EMC, by a factor of approx. 1.6-1.9 times.

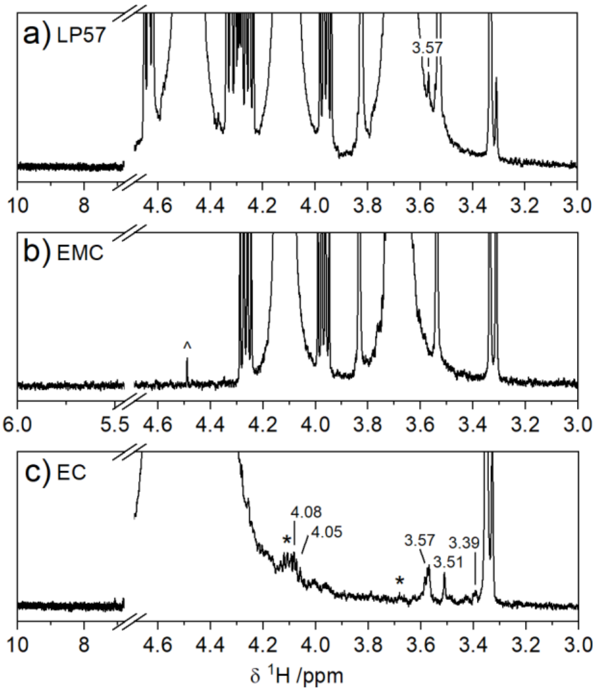

Figure S5. ^{1}H NMR of pristine electrolytes. (a) LP57. (b) 1.5 M $LiPF_6$ in ethyl methyl carbonate (EMC) – the chevron symbol marks a signal from a trace ethylene carbonate (EC) impurity. (c) 1.5 M $LiPF_6$ in EC – the asterisk marks signal from a trace EMC impurity. The signals labelled in (a) and (c) are attributed to hydrolysis products of EC. See assignments in Table S3.

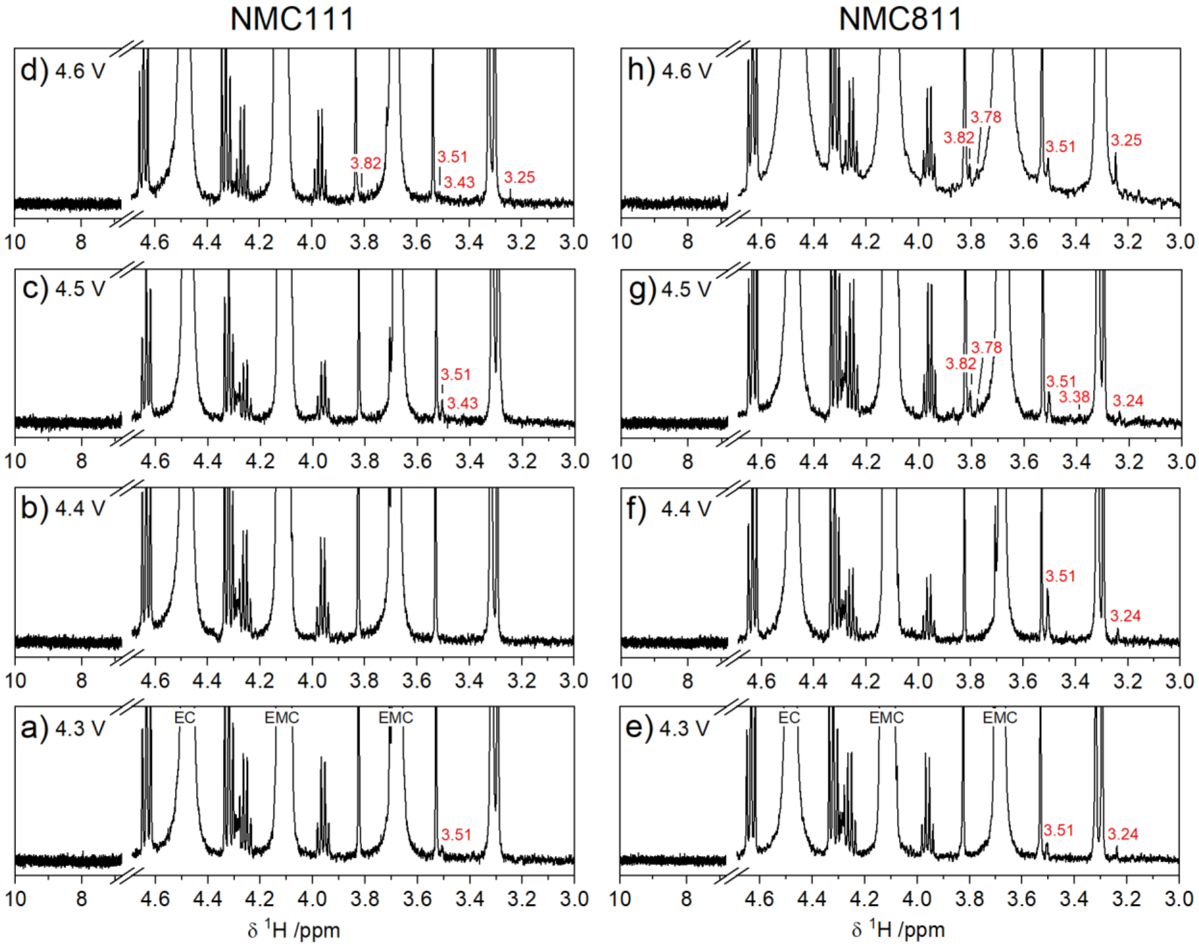

Figure S6. ^{1}H NMR of the electrolyte extracted from NMC/LTO cells with LP57 electrolyte for NMC111 (a-d) and NMC811 (e-h) after a C/20 charge to (a, e) 4.3, (b, f) 4.4, (c, g) 4.5, or (d, h) 4.6 V, 60 h voltage hold (VH), and C/20 discharge. Signals of EC and EMC are annotated in (a). The chemical shift labels in red correspond to signals that arise from electrolyte degradation.

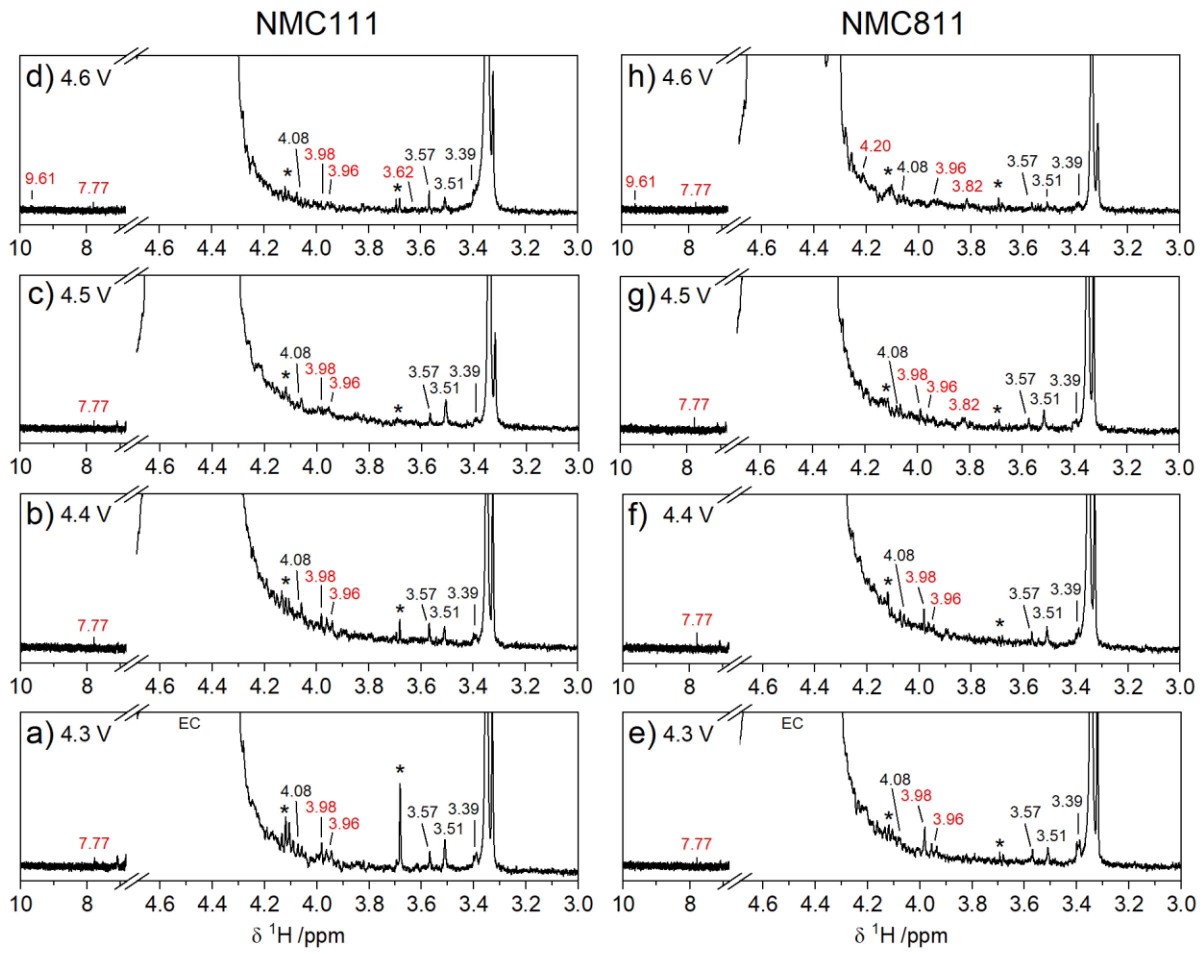

Figure S7. ^{1}H NMR of the electrolyte extracted from NMC/LTO cells with 1.5 M $LiPF_6$ in ethylene carbonate (EC) electrolyte for NMC111 (a-d) and NMC811 (e-h) after a C/20 charge to (a, e) 4.3, (b, f) 4.4, (c, g) 4.5, or (d, h) 4.6 V, 60 h voltage hold (VH), and C/20 discharge. Signals of EC are annotated in (a). Signals of a trace EMC impurity are marked with an asterisk. The chemical shift labels in black are also present in the pristine electrolyte, while red correspond to signals that arise from electrolyte degradation.

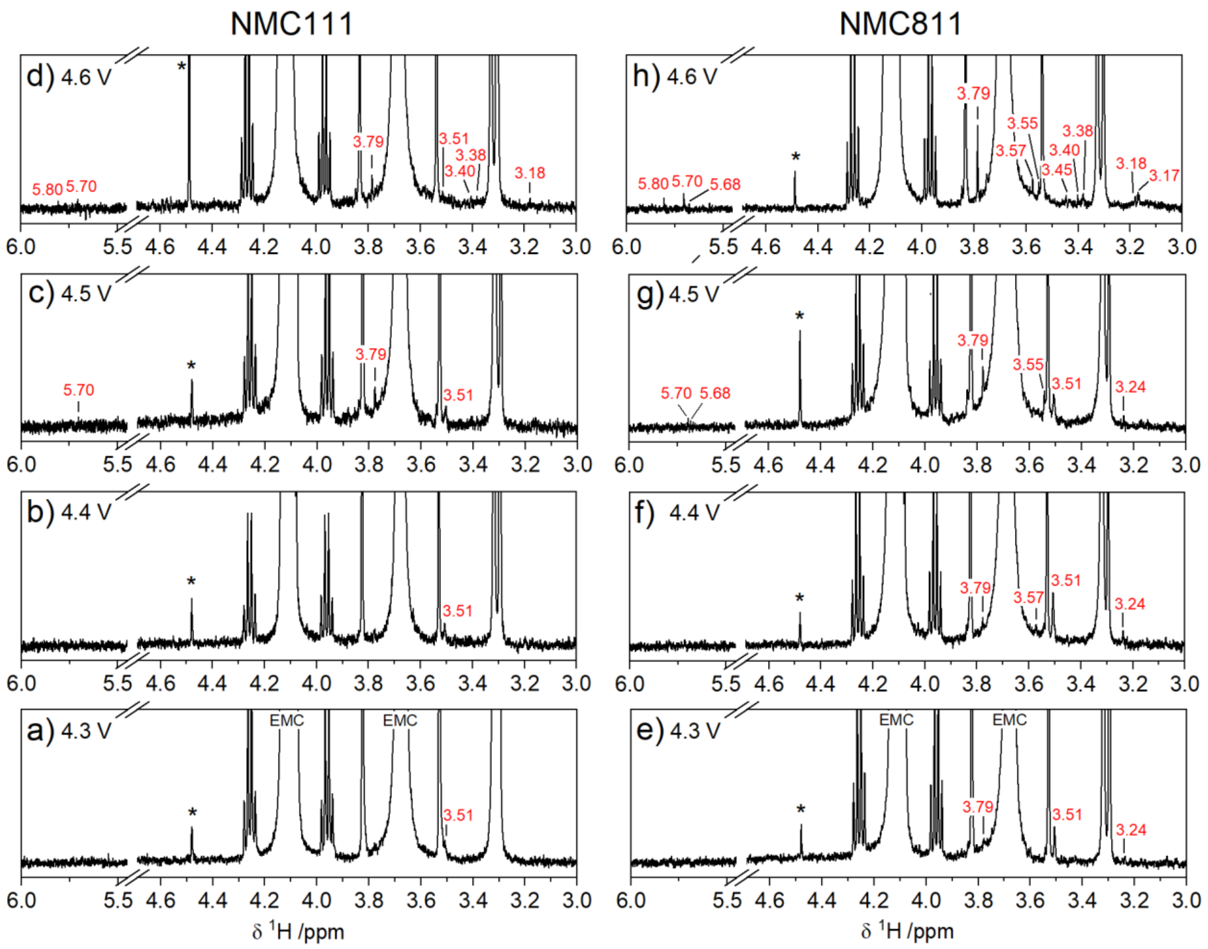

Figure S8. ¹H NMR of the electrolyte extracted from NMC/LTO cells with 1.5 M $LiPF_6$ in ethyl methyl carbonate (EMC) electrolyte for NMC111 (a-d) and NMC811 (e-h) after a C/20 charge to (a, e) 4.3, (b, f) 4.4, (c, g) 4.5, or (d, h) 4.6 V, 60 h voltage hold (VH), and C/20 discharge. Signals of EMC are annotated in (a). Signals of a trace EC impurity are marked with an asterisk. The chemical shift labels in red correspond to signals that arise from electrolyte degradation.

Table S3. Summary of observed chemical shifts in ^{1}H NMR spectra of pristine and cycled electrolytes and the corresponding assignments.[5–12] The J-coupling multiplicity is indicated in brackets; weak or minor peaks, where the J-coupling is less clear, are also indicated. Details of these assignments are discussed in detail in our recent work.[3]

chemical shift /ppm	assignment
4.48 (s)	ethylene carbonate (EC)
4.12 (q, $^{3}J_{H\text{-}H}$ = 7.1 Hz)	ethyl methyl carbonate (EMC)
3.69 (s)	EMC
1.21 (t, $^{3}J_{H\text{-}H}$ = 7.1 Hz)	EMC
9.61 (weak)	aldehyde RCHO (e.g. formaldehyde, acetaldehyde, glyoxal)
7.77 (s)	vinylene carbonate (VC)
5.80 (s)	acetal $RCH(OR)_2$ (e.g. methanediol, 1,1-ethanediol, methoxymethanol, and 1-methoxyethanol)
5.70 (s)	acetal
5.68 (s)	acetal
4.20 (m)	poly-ethylene oxide (EO) based oligomers $ROCOOCH_2CH_2OR'$
3.78-3.82 (s or m)	poly-EO based oligomers
3.38-3.62 (several m)	poly-EO based oligomers
3.24 (s)	poly-EO based oligomers
3.38-3.39 (m)	ethylene glycol
4.08 (t, $^{3}J_{H\text{-}H}$ = 4.7 Hz)	lithium ethylene monocarbonate (LEMC)
3.57 (t, $^{3}J_{H\text{-}H}$ = 4.7 Hz)	LEMC
3.98 (s)	oxyfluorophosphate salts
3.96 (d, $^{3}J_{P\text{-}H}$ = 10.1 Hz)	$OPF_2(OCH_3)$
3.34 (s)	water
3.32 (s)	DMSO impurity
3.18 (d, $^{3}J_{H\text{-}H}$ = 5.5 Hz)	methanol

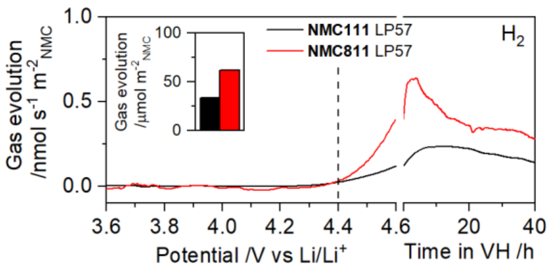

Figure S9. Evolution of H_2 from the OEMS channel m/z = 2, normalized to the NMC surface area, during a C/20 charge and 40 h VH at 4.6 V for NMC111 and 811 with LP57 electrolyte. Data is plotted versus NMC potential for the constant-current charge and versus time for the VH. Dashed vertical line shows the approximate onset potential of 4.4 V.

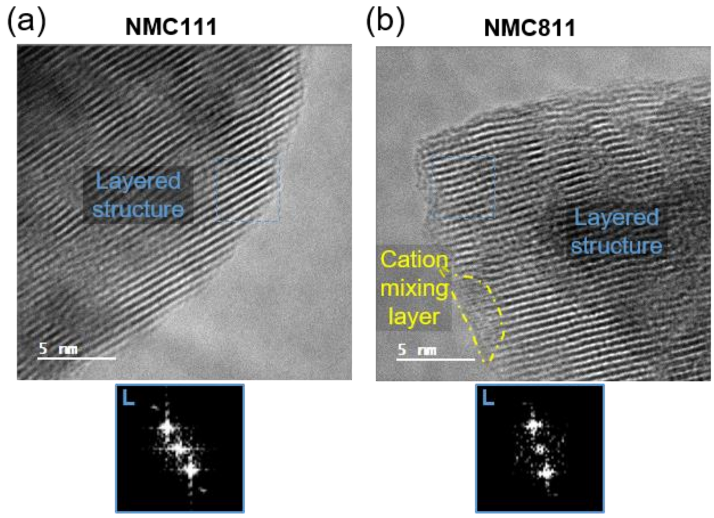

Figure S10. Pristine NMC interfacial structure. High resolution TEM images and corresponding fast Fourier transformation (FFT) images of (a) NMC111 and (b) NMC811 in the pristine state showing the layered structure. Pristine NMC811 also displays evidence of some layers with cation mixing between the Li and TM layers and dislocations/grain boundaries.

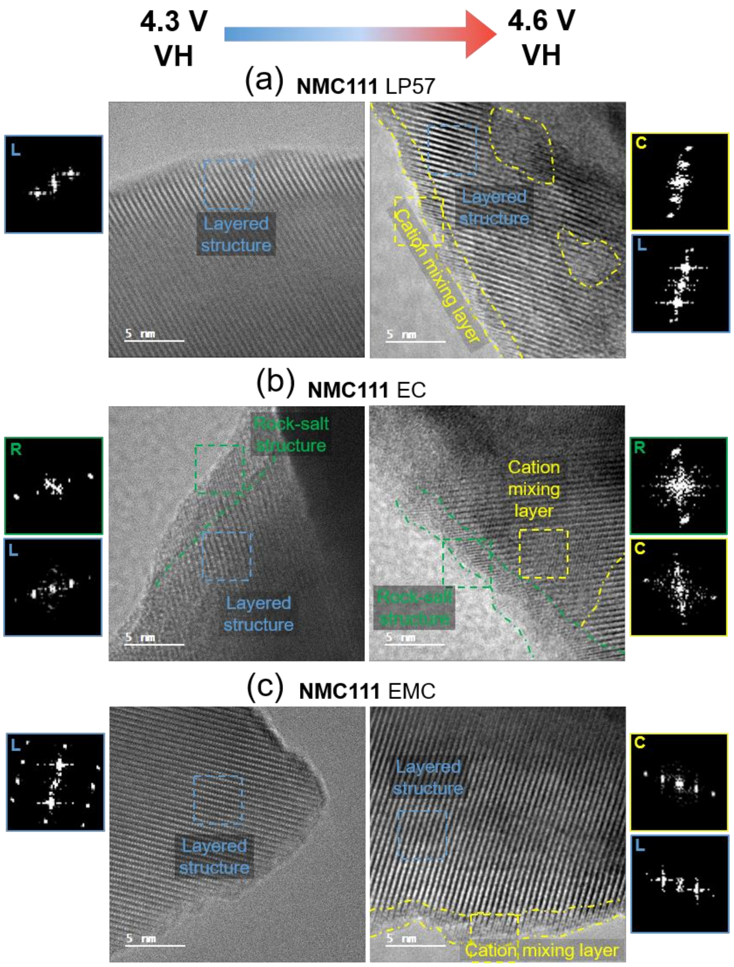

Figure S11. NMC111 interfacial structure. High resolution TEM images and corresponding fast Fourier transformation (FFT) images after the voltage holds (VHs) at (left) 4.3 V and (right) 4.6 V for NMC111 with (a) LP57 electrolyte, (b) 1.5 M $LiPF_6$ in EC, and (c) 1.5 M $LiPF_6$ in EMC. The dashed squares indicate where the FFTs are analyzed. The letters L, C, and R in the FFTs stand for layered structure, cation mixing layer, and rock-salt structure, respectively.

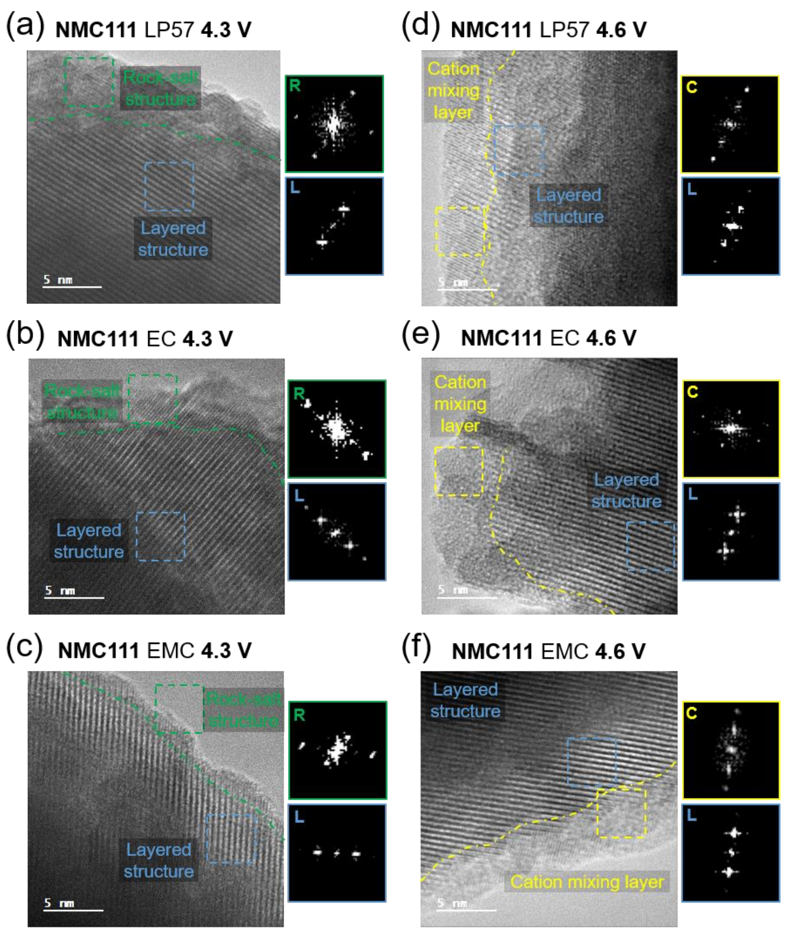

Figure S12. NMC111 interfacial structure – additional particles for conditions shown in Figure S11. High resolution TEM images and corresponding fast Fourier transformation (FFT) images after the voltage holds (VHs) at (a–c) 4.3 V and (d–e) 4.6 V for NMC111 with (a, d) LP57 electrolyte, (b, e) 1.5 M $LiPF_6$ in EC, and (c, f) 1.5 M $LiPF_6$ in EMC. The dashed squares indicate where the FFTs are analyzed. The letters L, C, and R in the FFTs stand for layered structure, cation mixing layer, and rock-salt structure, respectively.

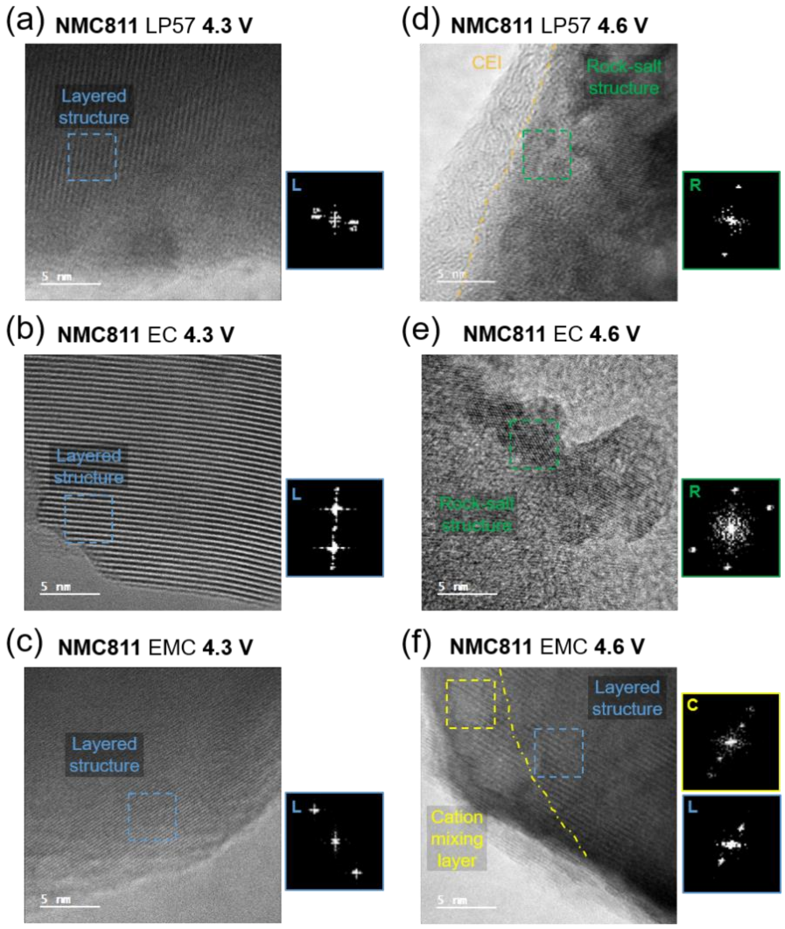

Figure S13. NMC811 interfacial structure – additional particles for conditions shown in Figure 2. High resolution TEM images and corresponding fast Fourier transformation (FFT) images after the voltage holds (VHs) at (a–c) 4.3 V and (d–e) 4.6 V for NMC811 with (a, d) LP57 electrolyte, (b, e) 1.5 M $LiPF_6$ in EC, and (c, f) 1.5 M $LiPF_6$ in EMC. The dashed squares indicate where the FFTs are analyzed. The letters L, C, and R in the FFTs stand for layered structure, cation mixing layer, and rock-salt structure, respectively.

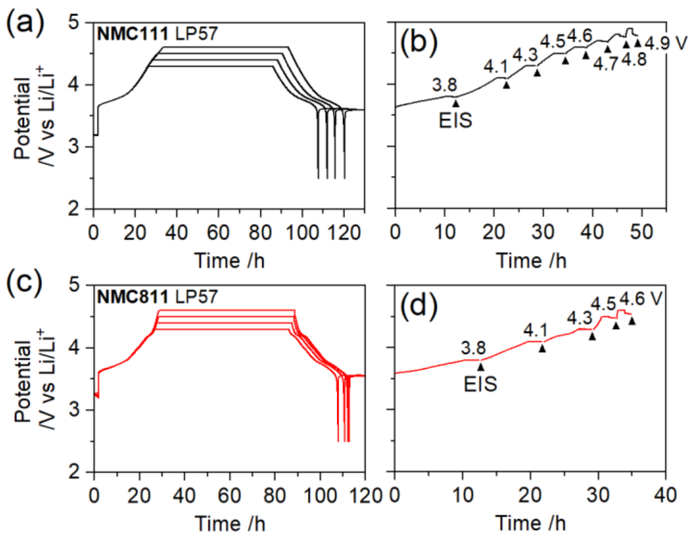

Figure S14. Representative NMC potential profiles for a three-electrode NMC/LTO cell with a Li metal reference electrode and LP57 electrolyte during (a, c) a C/20 charge to 4.3, 4.4, 4.5, or 4.6 V, 60 h voltage hold (VH), and C/20 discharge, and (b, d) the subsequent C/20 charge with intermittent potentiostatic holds, OCP periods, and electrochemical impedance spectroscopy (EIS) measurement at the NMC potentials indicated.

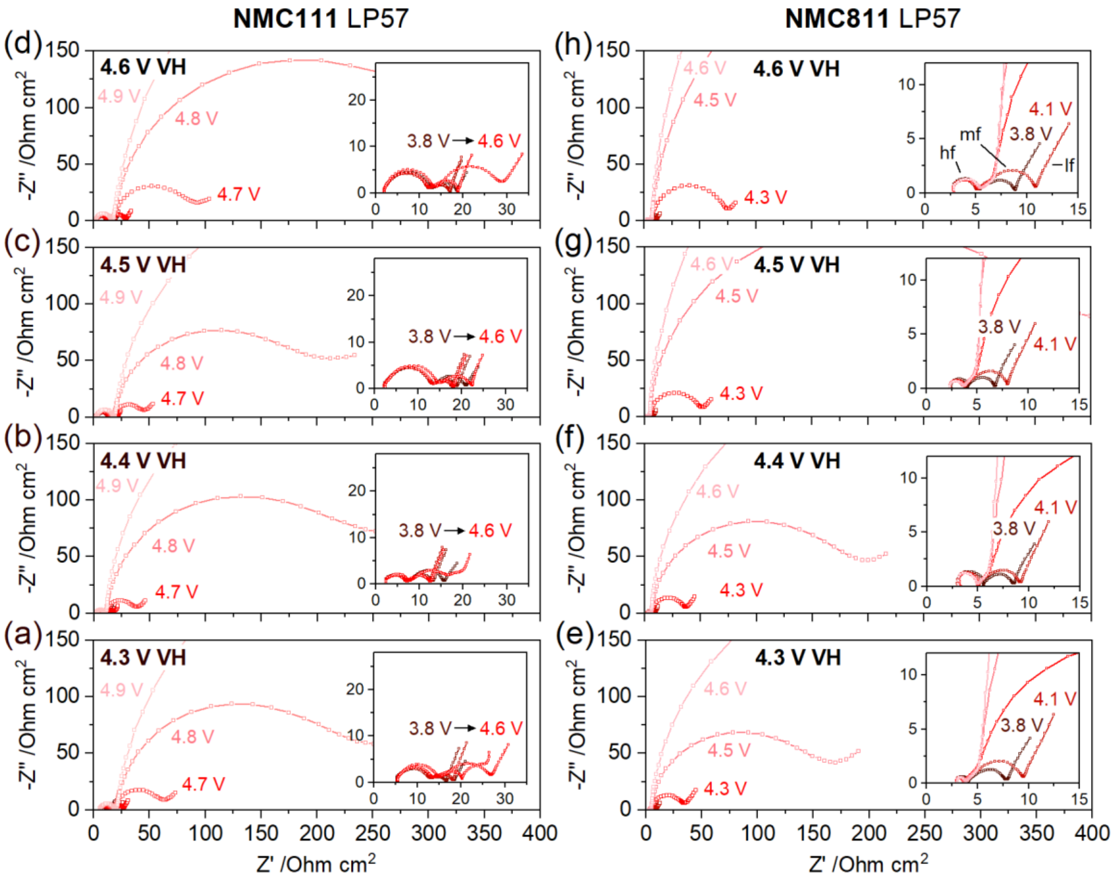

Figure S15. Nyquist impedance plots of the (a-d) NMC111 and NMC811 (e-h) cathode with LP57 electrolyte as a function of potential measured in a three-electrode NMC/LTO cell with a Li metal reference electrode after a C/20 charge to (a, e) 4.3, (b, f) 4.4, (c, g) 4.5, or (d, h) 4.6 V, 60 h voltage hold (VH), and C/20 discharge.

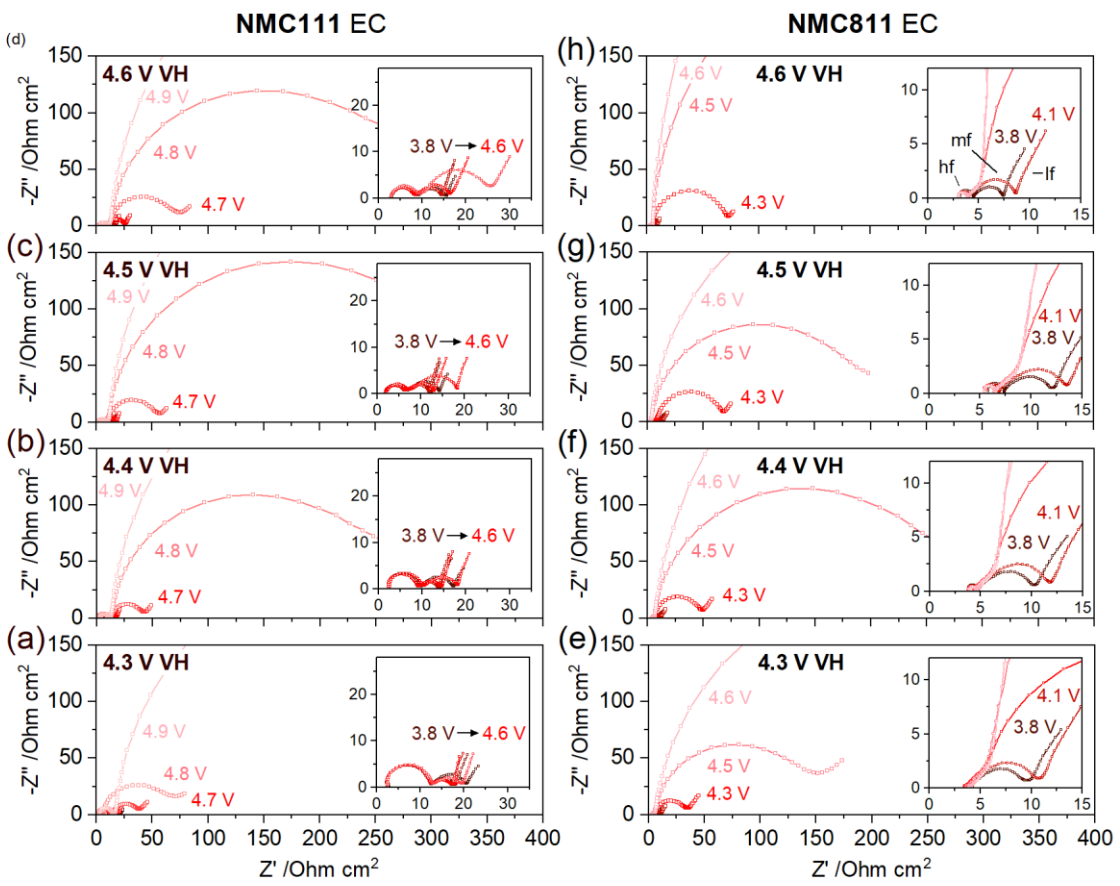

Figure S16. Nyquist impedance plots of the (a-d) NMC111 and NMC811 (e-h) cathode with 1.5 M $LiPF_6$ in ethylene carbonate (EC) electrolyte as a function of potential measured in a three-electrode NMC/LTO cell with a Li metal reference electrode after a C/20 charge to (a, e) 4.3, (b, f) 4.4, (c, g) 4.5, or (d, h) 4.6 V, 60 h voltage hold (VH), and C/20 discharge.

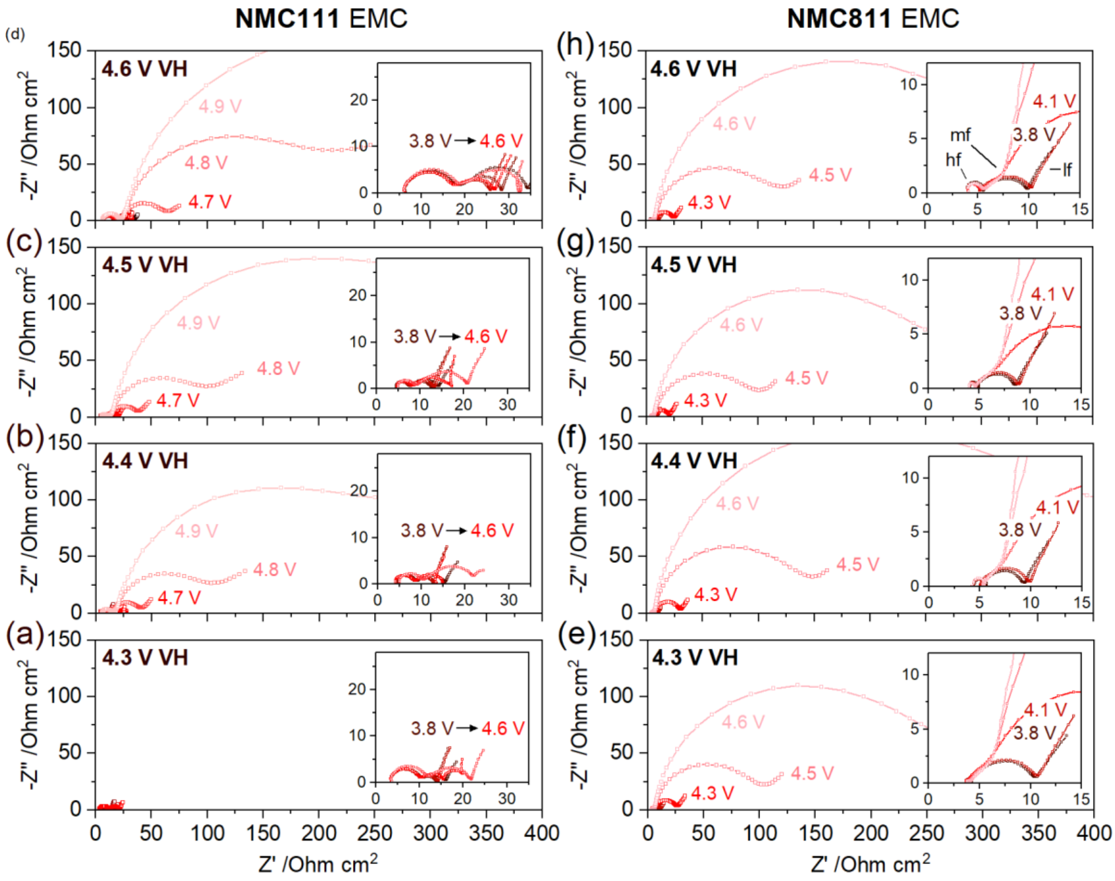

Figure S17. Nyquist impedance plots of the (a-d) NMC111 and NMC811 (e-h) cathode with 1.5 M $LiPF_6$ in ethyl methyl carbonate (EMC) electrolyte as a function of potential measured in a three-electrode NMC/LTO cell with a Li metal reference electrode after a C/20 charge to (a, e) 4.3, (b, f) 4.4, (c, g) 4.5, or (d, h) 4.6 V, 60 h voltage hold (VH), and C/20 discharge.

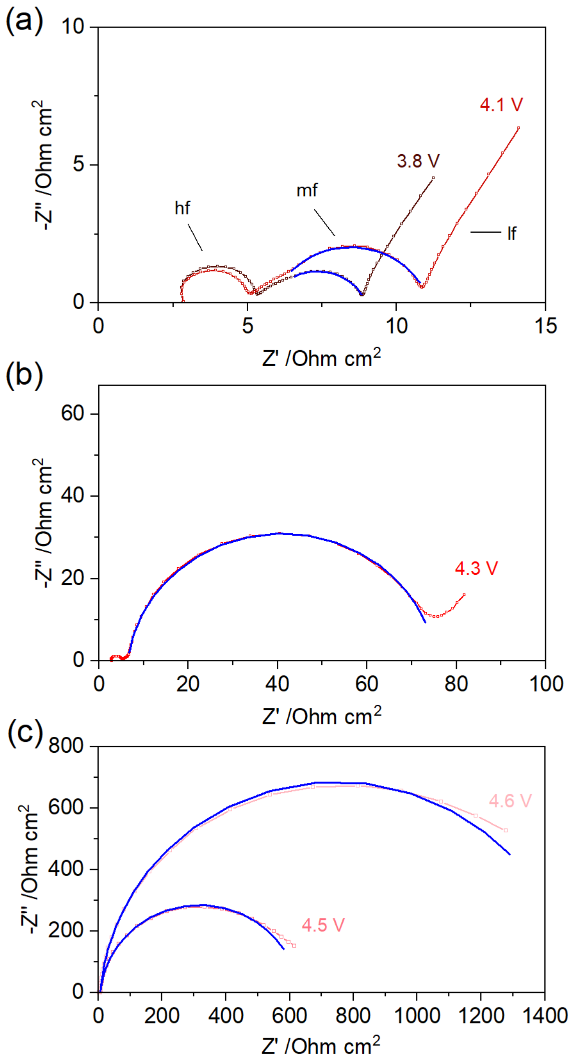

Figure S18. Exemplary Nyquist impedance plots (red) of the NMC cathode as a function of potential – (a) 3.8 and 4.1 V, (b) 4.3 V, and (c) 4.5 and 4.6 V – measured in a three-electrode NMC/LTO cell with a Li metal reference electrode. Data shown was collected with a NMC811 cathode and LP57 electrolyte. The high frequency semicircle (hf), mid-frequency semicircle (mf), and Warburg impedance tail at low frequencies (lf) are labelled. The mid-frequency semicircle was fit using a simple electrochemical equivalent circuit composed of a resistor (R_1), for horizontal displacement of the semicircle, in series with a resistor (R_2) and a constant phase element (CPE) in parallel, to describe the electrolyte-oxide interface resistance. The fit to the mid-frequency semicircle data is shown by a blue line through the red colored data points.

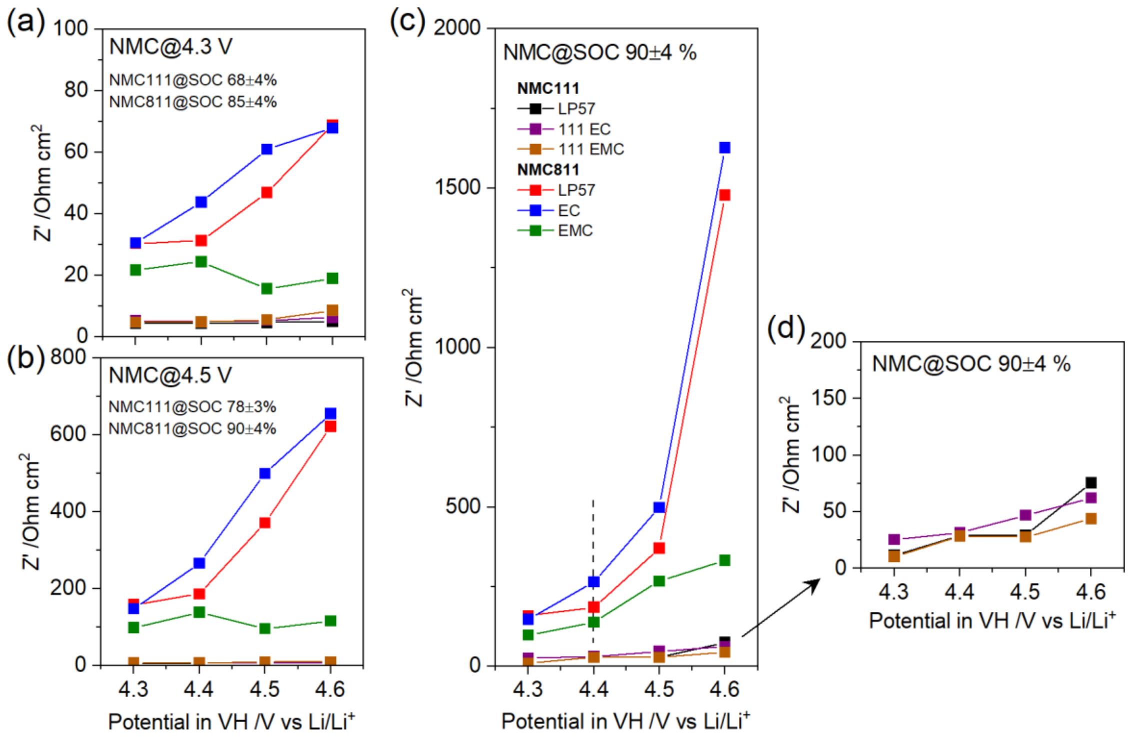

Figure S19. Charge transfer resistance (CTR) from electrochemical impedance spectroscopy (EIS) as a function of NMC potential in the VH, measured at an NMC (a) potential of 4.3 V vs. Li/Li^+, (b) potential of 4.5 V vs. Li/Li^+, and (c–d) state of charge (SOC) of 90±4 %. The CTR was extracted by fitting a simplified equivalent circuit to the data – see Figure S18 for details. In (a–b) the SOC of NMC111 and NMC811 are indicated. In (a–c) data for NMC111 and 811 are shown on the same axis for comparison. Dashed vertical line in (c) indicates the gas evolution onset potential at 4.4 V for NMC811. In (d) data for NMC111 from (c) is shown on a zoomed y-axis.

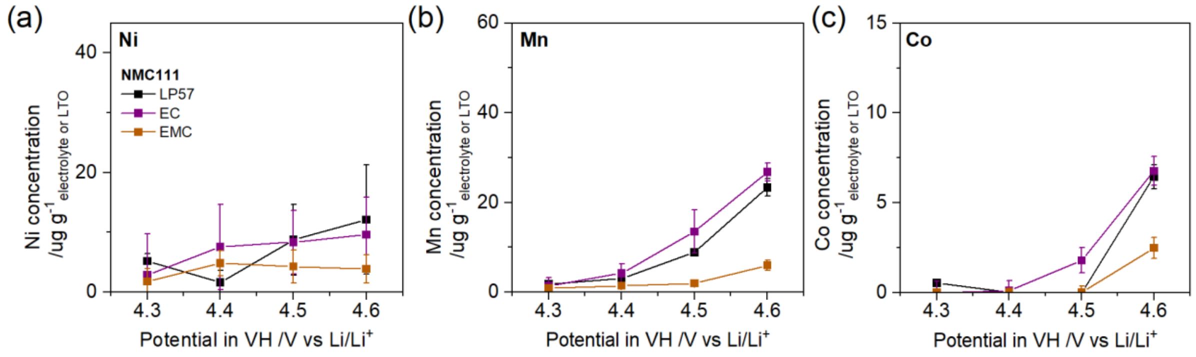

Figure S20. Transition metal dissolution/deposition with NMC111 cathode and electrolytes LP57, 1.5 M $LiPF_6$ in ethylene carbonate (EC), and 1.5 M $LiPF_6$ in ethyl methyl carbonate (EMC). Concentration of (a) Ni, (b) Mn, and (c) Co dissolved in the electrolyte and deposited on LTO electrodes, extracted after the VHs in the discharged state.

References

(1) Li, W.; Dolocan, A.; Li, J.; Xie, Q.; Manthiram, A. Ethylene Carbonate-Free Electrolytes for High-Nickel Layered Oxide Cathodes in Lithium-Ion Batteries. *Adv. Energy Mater.* **2019**, *9* (29), 1901152. https://doi.org/10.1002/aenm.201901152.

(2) Metzger, M.; Walke, P.; Solchenbach, S.; Salitra, G.; Aurbach, D.; Gasteiger, H. A. Evaluating the High-Voltage Stability of Conductive Carbon and Ethylene Carbonate with Various Lithium Salts. *J. Electrochem. Soc.* **2020**, *167* (16), 160522. https://doi.org/10.1149/1945-7111/abcabd.

(3) Dose, W. M.; Temprano, I.; Allen, J. P.; Björklund, E.; O'Keefe, C. A.; Li, W.; Mehdi, B. L.; Weatherup, R. S.; De Volder, M. F. L.; Grey, C. P. Electrolyte Reactivity at the Charged Ni-Rich Cathode Interface and Degradation in Li-Ion Batteries. *ACS Appl. Mater. Interfaces* **2022**, *14* (11), 13206–13222. https://doi.org/10.1021/acsami.1c22812.

(4) Jung, R.; Metzger, M.; Maglia, F.; Stinner, C.; Gasteiger, H. A. Oxygen Release and Its Effect on the Cycling Stability of $LiNi_xMn_yCo_zO_2$ (NMC) Cathode Materials for Li-Ion Batteries. *J. Electrochem. Soc.* **2017**, *164* (7), A1361–A1377. https://doi.org/10.1149/2.0021707jes.

(5) Fulmer, G. R.; Miller, A. J. M.; Sherden, N. H.; Gottlieb, H. E.; Nudelman, A.; Stoltz, B. M.; Bercaw, J. E.; Goldberg, K. I. NMR Chemical Shifts of Trace Impurities: Common Laboratory Solvents, Organics, and Gases in Deuterated Solvents Relevant to the Organometallic Chemist. *Organometallics* **2010**, *29* (9), 2176–2179. https://doi.org/10.1021/om100106e.

(6) Jin, Y.; Kneusels, N. J. H.; Grey, C. P. NMR Study of the Degradation Products of Ethylene Carbonate in Silicon-Lithium Ion Batteries. *J. Phys. Chem. Lett.* **2019**, *10* (20), 6345–6350. https://doi.org/10.1021/acs.jpclett.9b02454.

(7) Rinkel, B. L. D.; Hall, D. S.; Temprano, I.; Grey, C. P. Electrolyte Oxidation Pathways in Lithium-Ion Batteries. *J. Am. Chem. Soc.* **2020**, *142* (35), 15058–15074. https://doi.org/10.1021/jacs.0c06363.

(8) Dupré, N.; Cuisinier, M.; Guyomard, D. Electrode/Electrolyte Interface Studies in Lithium Batteries Using NMR. *Electrochem. Soc. Interface* **2011**, *20* (3), 61–67.

https://doi.org/10.1149/2.F06113if.

(9) Wiemers-Meyer, S.; Winter, M.; Nowak, S. Mechanistic Insights into Lithium Ion Battery Electrolyte Degradation-a Quantitative NMR Study. *Phys. Chem. Chem. Phys.* **2016**, *18* (38), 26595–26601. https://doi.org/10.1039/c6cp05276b.

(10) Yu, Y.; Karayaylali, P.; Katayama, Y.; Giordano, L.; Gauthier, M.; Maglia, F.; Jung, R.; Lund, I.; Shao-Horn, Y. Coupled $LiPF_6$ Decomposition and Carbonate Dehydrogenation Enhanced by Highly Covalent Metal Oxides in High-Energy Li-Ion Batteries. *J. Phys. Chem. C* **2018**, *122* (48), 27368–27382. https://doi.org/10.1021/acs.jpcc.8b07848.

(11) Abraham, R. J.; Byrne, J. J.; Griffiths, L.; Perez, M. ^{1}H Chemical Shifts in NMR: Part 23, the Effect of Dimethyl Sulphoxideversus Chloroform Solvent on ^{1}H Chemical Shifts. *Magn. Reson. Chem.* **2006**, *44* (5), 491–509. https://doi.org/10.1002/mrc.1747.

(12) Campion, C. L.; Li, W.; Lucht, B. L. Thermal Decomposition of $LiPF_6$-Based Electrolytes for Lithium-Ion Batteries. *J. Electrochem. Soc.* **2005**, *152* (12), A2327. https://doi.org/10.1149/1.2083267.
